# Supplementary material for: Air pollution disparities and equality assessments of US national decarbonization strategies
Source: Nat Commun. 2022 Dec 5;13:7488. doi: 10.1038/s41467-022-35098-4 (PMC9722701; doi:10.1038/s41467-022-35098-4)
Supplement: Supplementary file 1 — Supplementary Information [file 41467_2022_35098_MOESM1_ESM.pdf]

Air pollution disparities and equality assessments of US national decarbonization strategies

Supplemental Information (SI)

Teagan Goforth[1,*] and Destenie Nock[1,2,*]

1. Engineering and Public Policy, Carnegie Mellon University, USA
2. Civil and Environmental Engineering, Carnegie Mellon University, USA

Contents

A. Model description 2
B. Emission Rates 4
C. InMAP specifications 5
D. Generation 8
E. National Emissions 10
F. Regional Air Pollution Analysis 15
G. Air Pollution Equality Assessments 17

A. Model description

Figure S-1 presents the regions from Regional Energy Deployment System (ReEDS) that are used in the subnational analysis.

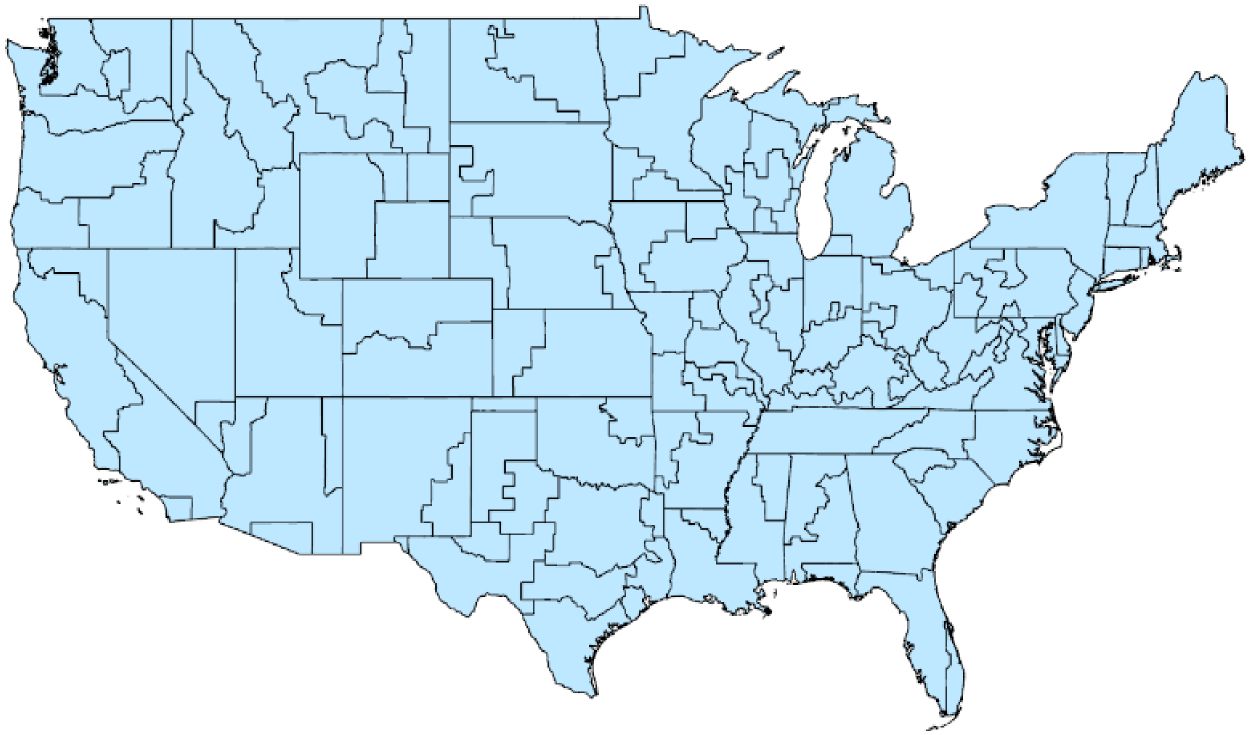

Figure S-1: ReEDS regions used in model evaluation (shapefile sourced from NREL ReEDS)[1].

Other than carbon policy implementations, no changes were made to the ReEDS' Mid_Case' inputs defined in the model inputs. The ReEDS' Mid_Case' uses the middle price assumptions for technologies. Relevant ReEDS 'Mid_Case' inputs and assumptions include: sequential solve (solve one year before continuing to the next), price assumptions for all technologies are from the US Energy Information Administration (EIA) 's Annual Energy Outlook (AEO) reference and mid-case, and current policy assumptions (AB32, CSAPR, wind Production Tax Credit (PTC), RGGI, state RPS). These cost, policy, and solve assumptions stay the same throughout all the scenarios. See the ReEDS documentation for a more thorough explanation of assumptions and inputs[1].

Table S-1 describes the carbon cap, national RPS, and low carbon technology mandates as input into ReEDS for each corresponding scenario.

Table S-1: Description of carbon cap (Mt CO_2/year), renewable portfolio standards (percent renewable energy generation per year), and low carbon technology mandates (percent low carbon technology generation per year) for each scenario.

	No carbon policies	Carbon cap (Mt CO_2/year)		Renewable energy generation mandate (%)			Low carbon generation mandate (%)	
Year	A	B	C	D	E	F	G	H
2010	-	4,808	4,808	0	0	0	0	0
2011	-	4,808	4,808	0	0	0	0	0
2012	-	4,808	4,808	0	0	0	0	0
2013	-	4,808	4,808	0	0	0	0	0
2014	-	4,808	4,808	0	0	0	0	0
2015	-	4,808	4,808	0	0	0	0	0
2016	-	4,808	4,808	0	0	0	0	0
2017	-	4,808	4,808	0	0	0	0	0
2018	-	4,808	4,808	0	0	0	0	0
2019	-	4,808	4,808	0	0	0	0	0
2020	-	1,341	1,341	20	20	20	20	20
2021	-	1,427	1,405	22	25	23	25	23
2022	-	1,329	1,258	24	31	25	31	25
2023	-	1,221	1,168	26	36	28	36	28
2024	-	1,116	1,092	28	41	31	41	31
2025	-	1,002	979	30	47	33	47	33
2026	-	973	889	32	52	36	52	36
2027	-	951	775	34	57	39	57	39
2028	-	928	649	36	63	41	63	41
2029	-	896	524	38	68	44	68	44
2030	-	844	464	40	73	47	73	47
2031	-	802	402	42	79	49	79	49
2032	-	766	323	44	84	52	84	52
2033	-	734	245	46	89	55	89	55
2034	-	700	178	48	95	57	95	57
2035	-	668	167	50	100	60	100	60
2036	-	637	160	52	100	63	100	63
2037	-	610	154	54	100	65	100	65
2038	-	586	146	56	100	68	100	68
2039	-	575	137	58	100	71	100	71
2040	-	582	126	60	100	73	100	73
2041	-	589	114	62	100	76	100	76
2042	-	594	103	64	100	79	100	79
2043	-	601	92	66	100	81	100	81

2044	-	608	80	68	100	84	100	84
2045	-	613	69	70	100	87	100	87
2046	-	619	57	72	100	89	100	89
2047	-	624	46	74	100	92	100	92
2048	-	629	34	76	100	95	100	95
2049	-	634	23	78	100	97	100	97
2050	-	639	11	80	100	100	100	100

B. Emission Rates

The emissions rate sources for each technology type included in the environmental sustainability analysis are shown in Table S-2.

Table S-2: Emission rates sources.

	Operating Emission Rates			
	CO_2eq.	NO_x	SO_2	$PM_{2.5}$
Biopower	1	1	1	
Solar photovoltaic	1	1	1	-
Concentrated solar power (CSP)	1	1	1	-
Onshore wind	1	1	1	-
Offshore wind	1	1	1	-
Nuclear	1	1	1	-
Natural gas combustion turbine (CT)	1	1	1	2
Natural gas combined cycle (CC)	1	1	1	2
Natural gas CCS	1	1	1	2
Hydropower	1	1	1	-
Geothermal	1	1	1	
Oil-Gas-Steam	1	1	1	2
Coal	1	1	1	2
IGCC	1	1	1	2
Coal CCS	1	1	1	2
Battery storage	1	1	1	-
Pumped hydropower	1	1	1	-

Table S-3 presents the heat rates for each fossil fuel plant used to estimate the emissions rate. Since most renewable technologies do not convert fuel to electricity, they do not have a heat rate (excluding biopower). We also assume renewable energy besides biopower does not emit anything in operation.

Table S-3: Heat rates for power plants in MMBtu/MWh and fuel rates in pounds (lb)/MMBtu.[1]

Technology	CO_2 fuel rate [lb/MMBtu]	NO_x fuel rate [lb/MMBtu]	SO_2 fuel rate [lb/MMBtu]	$PM_{2.5}$ fuel rate [lb/MMBtu]	Heat rate [MMBtu/MWh]
Coal	211	0.153	0.470	0.016	9.669
Cofire	179	0.130	0.411	0.029	10.112
Coal CCS	21.1	0.085	0.056	0.016	10.157
Coal IGCC	211	0.085	0.056	0.016	7.920
Gas CC	117	0.02	0.005	0.007	6.341
Gas CCS	11.7	0.02	0.005	0.007	7.505
Gas CT	117	0.15	0.015	0.007	9.36
Oil-Gas-Steam	137	0.172	0.299	0.116	10.648
Biopower	0	0	0.08	0.101	13.5
Nuclear	0	0	0	-	10.461

C. InMAP specifications

The Intervention Model for Air Pollution (InMAP) intakes emissions at the ReEDS level and area weights them across the ReEDS region to fit InMAP defined regions (1x1 km to 48x48 km squares). InMAP uses these emissions input to estimate the average annual concentration. While running, InMAP uses a reaction-advection-diffusion equation that estimates where air pollution ends up as ambient concentrations. The model uses a steady state formulation for each time step and continues to run until the air pollution concentrations reach steady state (the change in concentration is zero). Within each time step, each region accounts for the flux of new emissions and how pollution concentrations are affected by physical and chemical processes. Once the model reaches steady state, it outputs a shapefile with the annual average ambient concentrations for each region. [3]

InMAP uses area-weighting to distribute emissions at the ReEDS level (134 regions) to the InMAP level (squares with 1 to 48 km sides). Equations S-1a and S-1b display the two-step process for area-weighting[4]. In Equation S-1a, the areal weight for each InMAP region is calculated, where $W_{I,i}$ is the areal weight for the InMAP region i (in km^2), $A_{I,i}$ is the area of the InMAP region (in km^2), and $A_{R,j}$ is the area of the ReEDS region j. Equation S-1b calculates the areal weighted air pollution value in each InMAP region, where $E_{I,i}$ is the estimated air pollution

magnitude in each InMAP region and $Q_{R,j}$ is the PM, NO_x, or SO_2 air pollution (in kilograms) in each ReEDS region j.

$$W_{I,i} = \frac{A_{I,i}}{A_{R,j}} \qquad \text{(S-1a)}$$

$$E_{I,i} = W_{I,i} * Q_{R,j} \qquad \text{(S-1b)}$$

InMAP requires emissions inputs in shapefile form at the ReEDS region to perform this area weighting. Figure S-2(a-c) below shows the NO_x, SO_2, and PM emissions inputs, respectively (in metric tonnes) for InMAP, which show the total emissions in each region in 2020, 2035, and 2050.

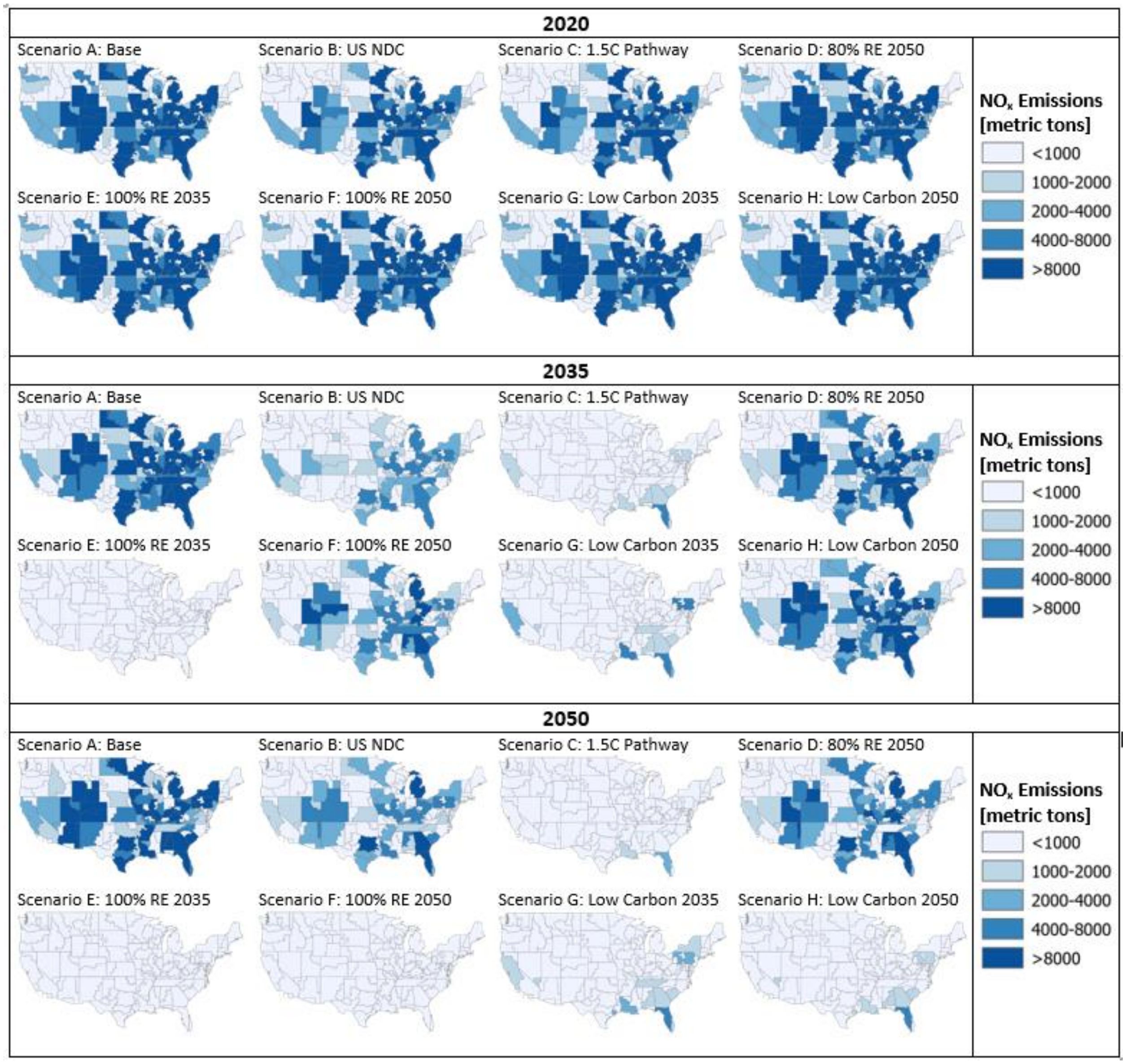

(a) NO_x

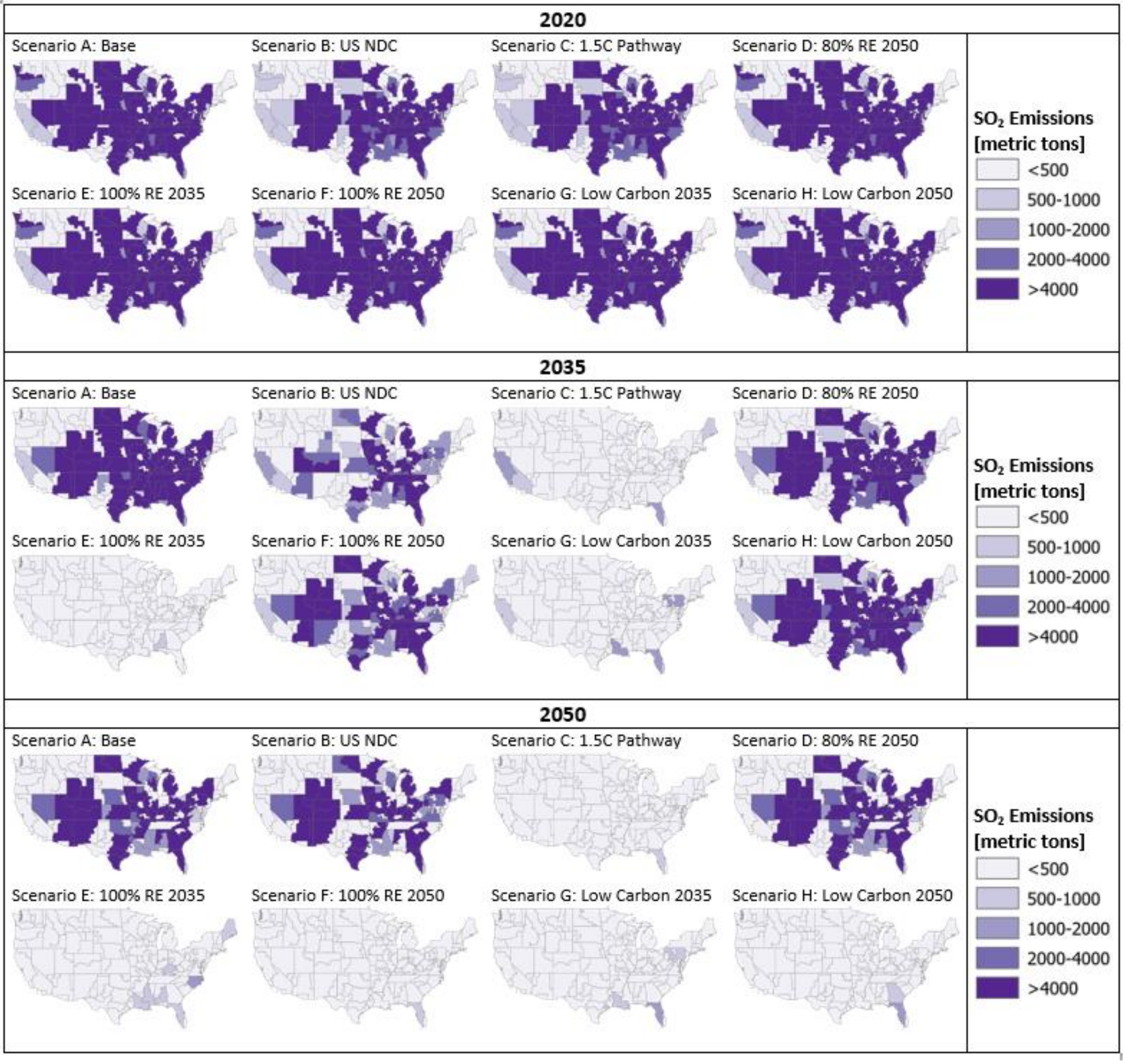
2020
Scenario A: Base
Scenario B: US NDC
Scenario C: 1.5C Pathway
Scenario D: 80% RE 2050
Scenario E: 100% RE 2035
Scenario F: 100% RE 2050
Scenario G: Low Carbon 2035
Scenario H: Low Carbon 2050
SO2 Emissions [metric tons]
<500
500-1000
1000-2000
2000-4000
>4000
2035
2050

(b) SO_2

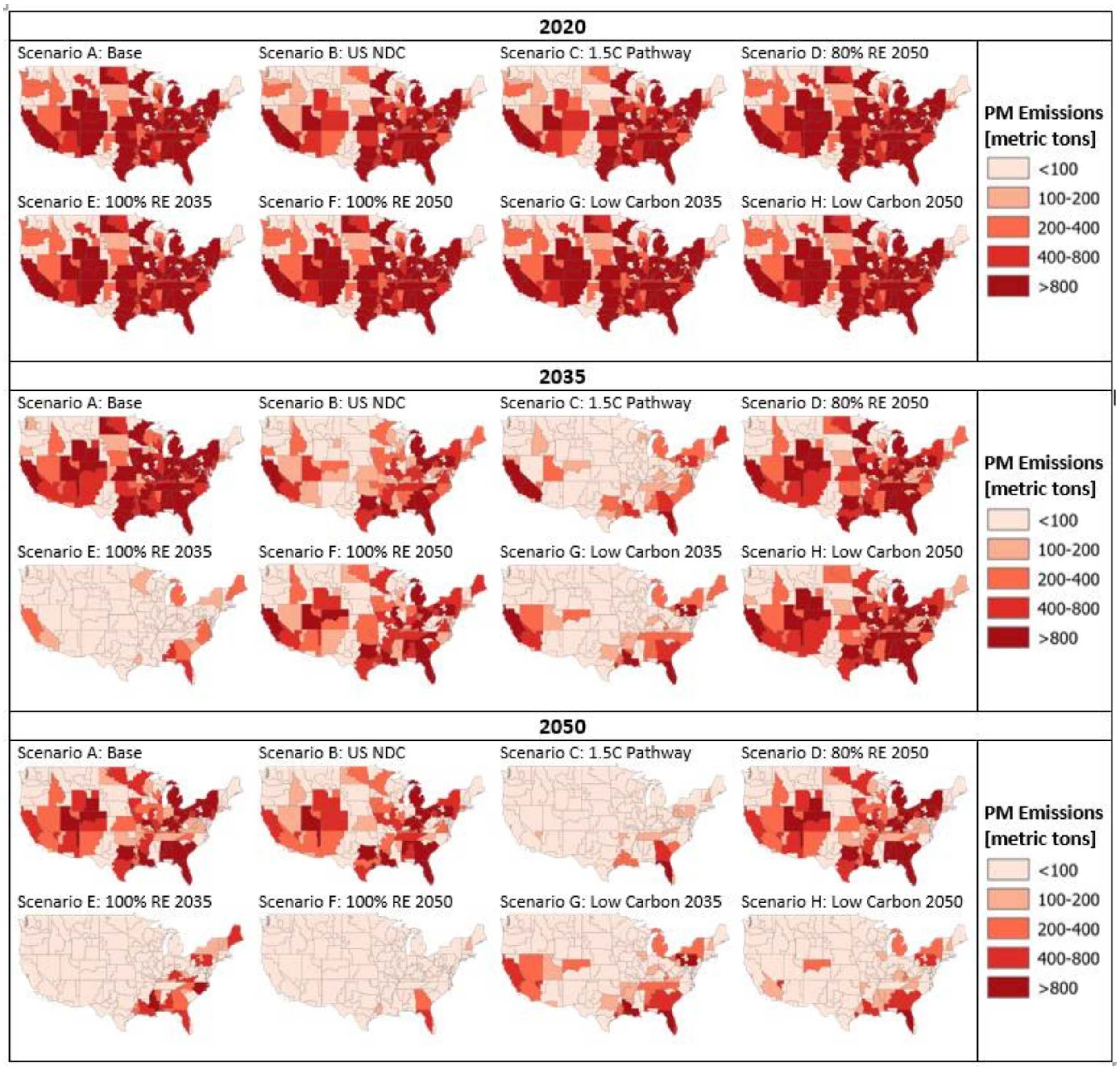

(c) $PM_{2.5}$

Figure S-2: Total (a) NO_x (b) SO_2 and (c) $PM_{2.5}$ emissions in each ReEDS region 2020, 2035, and 2050 for each scenario.

D. Generation

Table S-4 summarizes the percent of annual generation and the magnitude of generation for the main technologies across scenarios and by decade.

Table S-4: Summary of percent and magnitude of generation from main technologies by year and scenario.

		2010		2020		2030		2040		2050	
	Technology	%	PWh	%	PWh	%	PWh	%	PWh	%	PWh
A: Base	Coal	50.6	2.05	26.3	1.10	23.9	1.07	18.6	0.91	7.46	0.41
	Natural Gas	18.7	0.76	35.1	1.46	32.6	1.46	26.5	1.29	19.9	1.08
	Natural Gas CCS	0.0	0.00	0.0	0.00	0.0	0.00	0.0	0.00	0.0	0.00
	Nuclear	19.7	0.80	17.7	0.74	15.1	0.68	12.0	0.58	6.9	0.37
	Solar PV	0.1	0.00	3.6	0.15	7.9	0.35	14.1	0.69	20.9	1.14
	CSP	0.0	0.00	0.06	0.0	0.24	0.01	0.52	0.03	0.48	0.03
	Onshore Wind	2.6	0.11	8.4	0.35	10.6	0.48	19.1	0.93	33.8	1.83
	Offshore Wind	0.0	0.00	0.0	0.00	1.3	0.06	1.6	0.08	3.1	0.17
B: US NDC	Coal	50.6	2.05	16.2	0.67	5.5	0.25	1.7	0.08	4.7	0.25
	Natural Gas	18.7	0.76	40.3	1.68	35.2	1.58	27.5	1.35	19.7	1.07
	Natural Gas CCS	0.0	0.00	0.0	0.00	0.0	0.00	0.0	0.00	0.0	0.00
	Nuclear	19.7	0.80	17.7	0.74	15.0	0.68	11.9	0.58	6.9	0.37
	Solar PV	0.1	0.00	3.9	0.16	9.6	0.43	14.8	0.73	20.3	1.11
	CSP	0.0	0.00	0.1	0.00	0.2	0.01	0.8	0.04	0.7	0.04
	Onshore Wind	2.6	0.11	13.0	0.54	24.5	1.10	33.8	1.66	37.0	2.01
	Offshore Wind	0.0	0.00	0.0	0.00	1.3	0.06	1.6	0.08	3.1	0.17
C: 1.5C Pathway	Coal	50.6	2.05	16.2	0.67	0.2	0.01	0.0	0.00	0.0	0.00
	Natural Gas	18.7	0.76	40.3	1.68	26.8	1.21	6.1	0.30	0.2	0.01
	Natural Gas CCS	0.0	0.00	0.0	0.00	0.0	0.00	7.1	0.35	3.1	0.17
	Nuclear	19.7	0.80	0.74	17.7	0.68	15.0	11.5	0.58	5.5	0.30
	Solar PV	0.1	0.00	3.9	0.16	14.1	0.64	19.5	0.97	27.8	1.55
	CSP	0.0	0.00	0.1	0.00	0.2	0.01	1.2	0.06	1.4	0.08
	Onshore Wind	2.6	0.11	13.0	0.54	33.7	1.52	44.2	2.21	49.9	2.78
	Offshore Wind	0.0	0.00	0.0	0.00	1.2	0.06	1.7	0.09	4.2	0.23
D: 80% RE 2050	Coal	50.6	2.05	26.3	1.10	18.4	0.83	7.7	0.38	2.6	0.14
	Natural Gas	18.7	0.76	35.1	1.46	26.5	1.19	20.2	0.99	10.8	0.60
	Natural Gas CCS	0.0	0.00	0.0	0.00	0.0	0.00	0.0	0.00	0.0	0.00
	Nuclear	19.7	0.80	17.7	0.74	15.0	0.68	11.9	0.58	6.3	0.35
	Solar PV	0.1	0.00	3.6	0.15	10.2	0.46	16.5	0.81	22.1	1.22
	CSP	0.0	0.00	0.1	0.00	0.3	0.01	0.7	0.03	0.6	0.03
	Onshore Wind	2.6	0.11	8.4	0.35	19.8	0.89	33.4	1.64	44.9	2.49
	Offshore Wind	0.0	0.00	0.0	0.00	1.3	0.06	1.6	0.08	4.4	0.24
E: 100% RE 2035	Coal	50.6	2.05	26.3	1.10	0.9	0.04	0.0	0.00	0.0	0.00
	Natural Gas	18.7	0.76	35.1	1.46	15.83	0.73	0.0	0.00	0.0	0.00
	Natural Gas CCS	0.0	0.00	0.0	0.00	0.0	0.00	0.0	0.00	0.0	0.00
	Nuclear	19.7	0.80	17.7	0.74	9.7	0.44	0.0	0.00	0.0	0.00
	Solar PV	0.1	0.00	3.6	0.15	16.8	0.77	35.0	1.84	31.0	1.78
	CSP	0.0	0.00	0.1	0.00	0.4	0.02	9.6	0.51	9.8	0.56
	Onshore Wind	2.6	0.11	8.4	0.35	45.9	2.10	45.3	2.39	48.4	2.78
	Offshore Wind	0.0	0.00	0.0	0.00	1.2	0.05	1.5	0.08	3.5	0.20

F: 100% RE 2050	Coal	50.6	2.05	26.3	1.10	13.0	0.59	2.1	0.11	0.0	0.00
	Natural Gas	18.7	0.76	35.1	1.46	25.2	1.14	13.7	0.68		
	Natural Gas CCS	0.0	0.00	0.0	0.00	0.0	0.00	0.0	0.00	0.0	0.00
	Nuclear	19.7	0.80	17.7	0.74	15.0	0.68	10.7	0.53	0.0	0.00
	Solar PV	0.1	0.00	3.6	0.15	10.8	0.49	16.8	0.83	35.9	2.05
	CSP	0.0	0.00	0.1	0.00	0.3	0.01	1.1	0.05	3.3	0.19
	Onshore Wind	2.6	0.11	8.4	0.35	25.7	1.16	45.7	2.26	49.3	2.81
	Offshore Wind	0.0	0.00	0.0	0.00	1.3	0.06	1.7	0.08	4.6	0.26
G: Low Carbon 2035	Coal	50.6	2.05	26.3	1.10	4.8	0.22	0.0	0.00	0.0	0.00
	Natural Gas	18.7	0.76	35.1	1.46	21.8	0.98	0.0	0.00	0.0	0.00
	Natural Gas CCS	0.0	0.00	0.0	0.00	0.2	0.01	13.7	0.69	11.0	0.61
	Nuclear	19.7	0.80	17.7	0.74	15.0	0.68	11.4	0.58	6.5	0.36
	Solar PV	0.1	0.00	3.6	0.15	14.0	0.63	22.7	1.15	23.7	1.32
	CSP	0.0	0.00	0.1	0.00	0.2	0.01	1.3	0.07	1.2	0.07
	Onshore Wind	2.6	0.11	8.4	0.35	34.2	1.54	40.1	2.03	45.1	2.52
	Offshore Wind	0.0	0.00	0.0	0.00	1.2	0.06	1.6	0.08	3.8	0.02
H: Low Carbon 2050	Coal	50.6	2.05	26.3	1.10	22.3	1.00	6.1	0.30	0.0	0.00
	Natural Gas	18.7	0.76	35.1	1.46	31.0	1.39	20.5	1.00	0.0	0.00
	Natural Gas CCS	0.0	0.00	0.0	0.00	0.0	0.00	0.0	0.00	6.7	0.37
	Nuclear	19.7	0.80	17.7	0.74	15.1	0.68	11.9	0.58	5.7	0.32
	Solar PV	0.1	0.00	3.6	0.15	7.8	0.35	15.5	0.76	26.5	1.48
	CSP	0.0	0.00	0.1	0.00	0.3	0.01	0.8	0.04	0.8	0.05
	Onshore Wind	2.6	0.11	8.4	0.35	13.9	0.62	35.8	1.75	47.7	2.67
	Offshore Wind	0.0	0.00	0.0	0.00	1.3	0.06	1.6	0.08	4.4	0.24

E. National Emissions

The operating emissions are a function of the fuel emissions rate (e_f) in pounds/MMBtu, heat rate (H) in MMBtu/MWh, generation ($g_{n,t}$) for each technology (n) in each year (t) in MWh, and pounds to grams conversion (α) (Equation S-1). The total operating emissions are the sum of emissions from each technology (n).

$$E_O = \sum_{i=1}^{n} g_n e_{f,n} H_n \alpha \qquad \text{(S-2)}$$

Figures S-3 – S-6 display the national operating emission by technology 2010 – 2050 across each scenario and for each pollutant. For operating emissions, natural gas and coal technologies contribute most emissions. For SO_2 and $PM_{2.5}$ emissions, coal technologies contribute to 99% of operating emissions across all technologies.

Figure S-7 displays the national emissions ratio between $PM_{2.5}$ emissions and total generation (in kg/MWh). We find that the 1.5 C decarbonization pathway (Scenario C) often has the lowest or second lowest ratio over the entire modeling horizon. This most likely stems from Scenario C (1.5 C decarbonization pathway) retiring the entire coal fleet in the same period as Scenario E (100% RE by 2035). The scenarios with 100% RE generation achieve the lowest national emissions ratios in their mandate years.

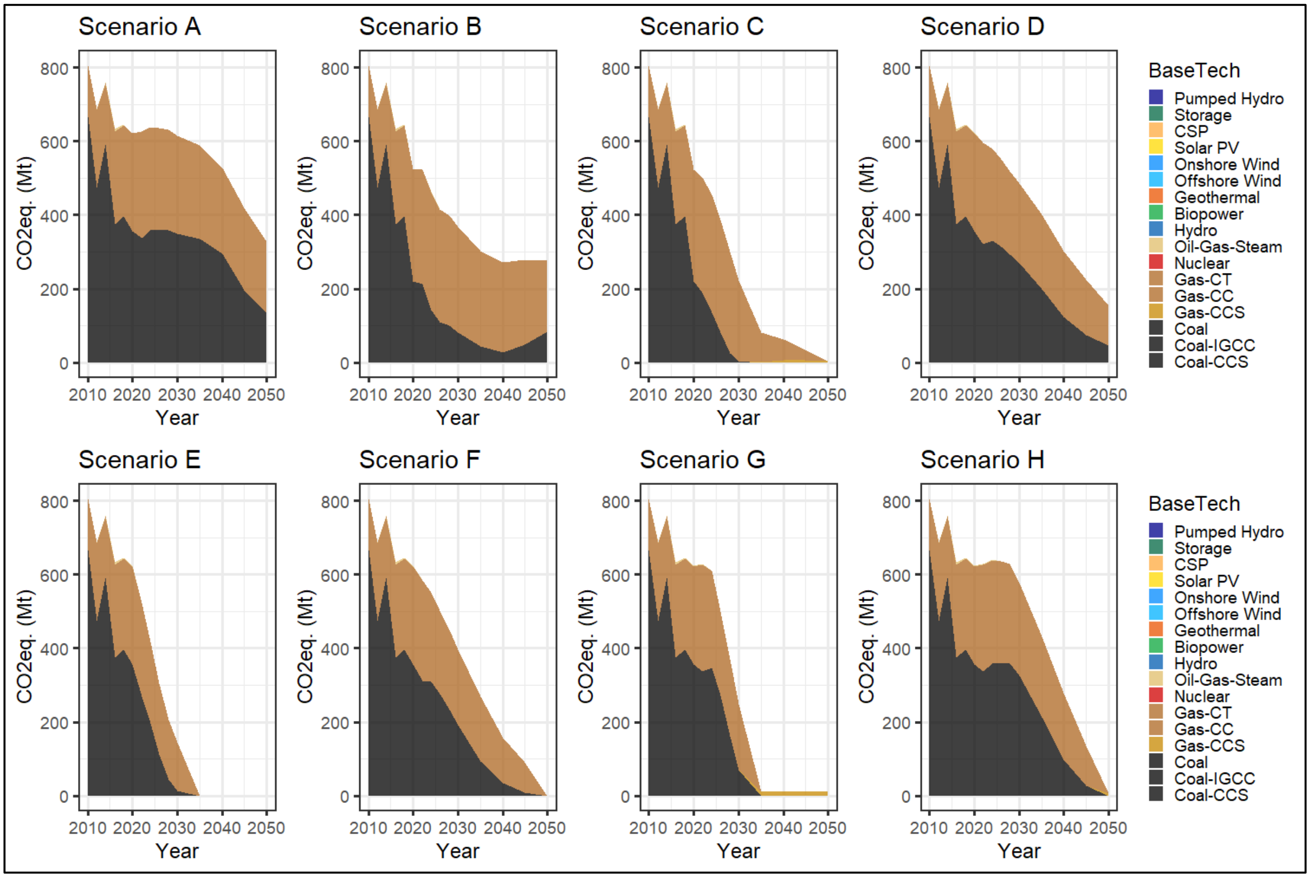

Figure S-3: National operating CO_2 emissions by technology 2010 – 2050.

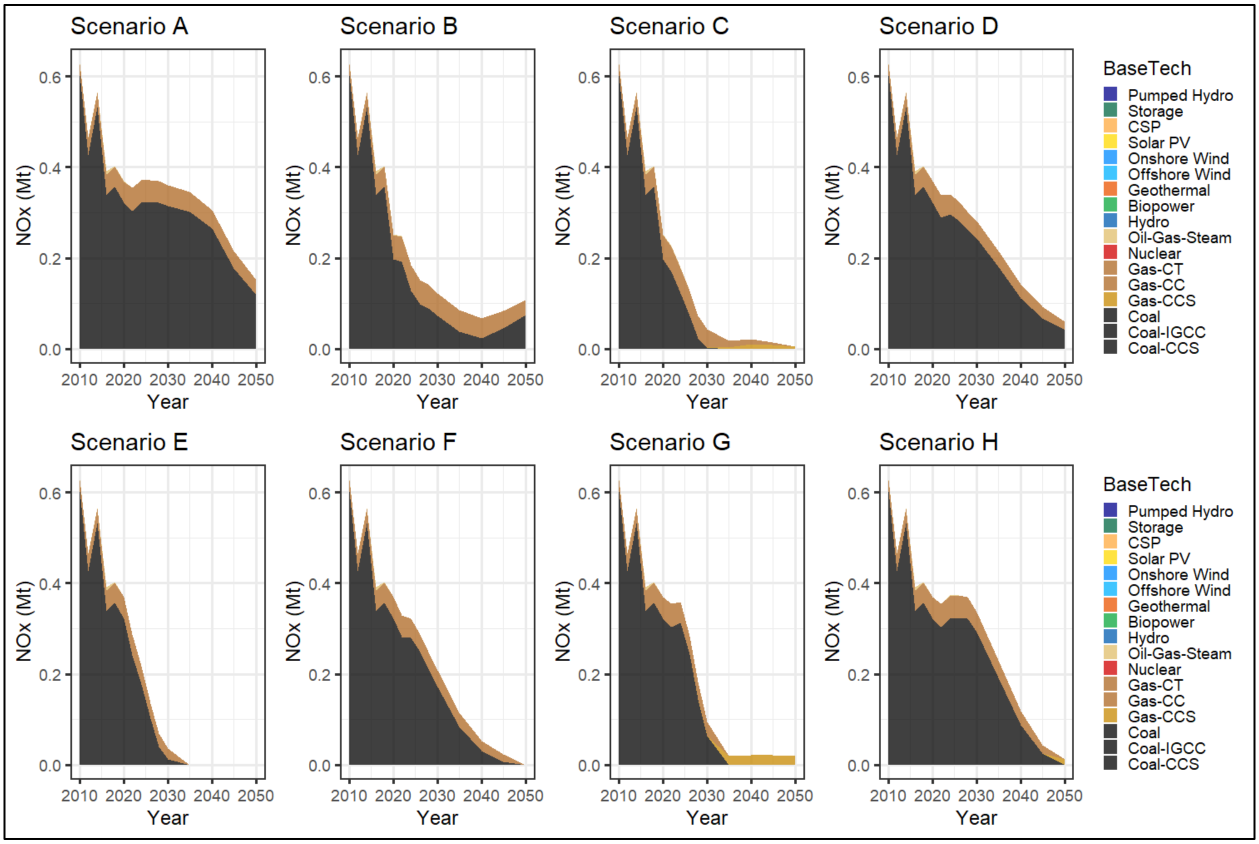

Figure S-4: National operating NO_x emissions by technology 2010 – 2050.

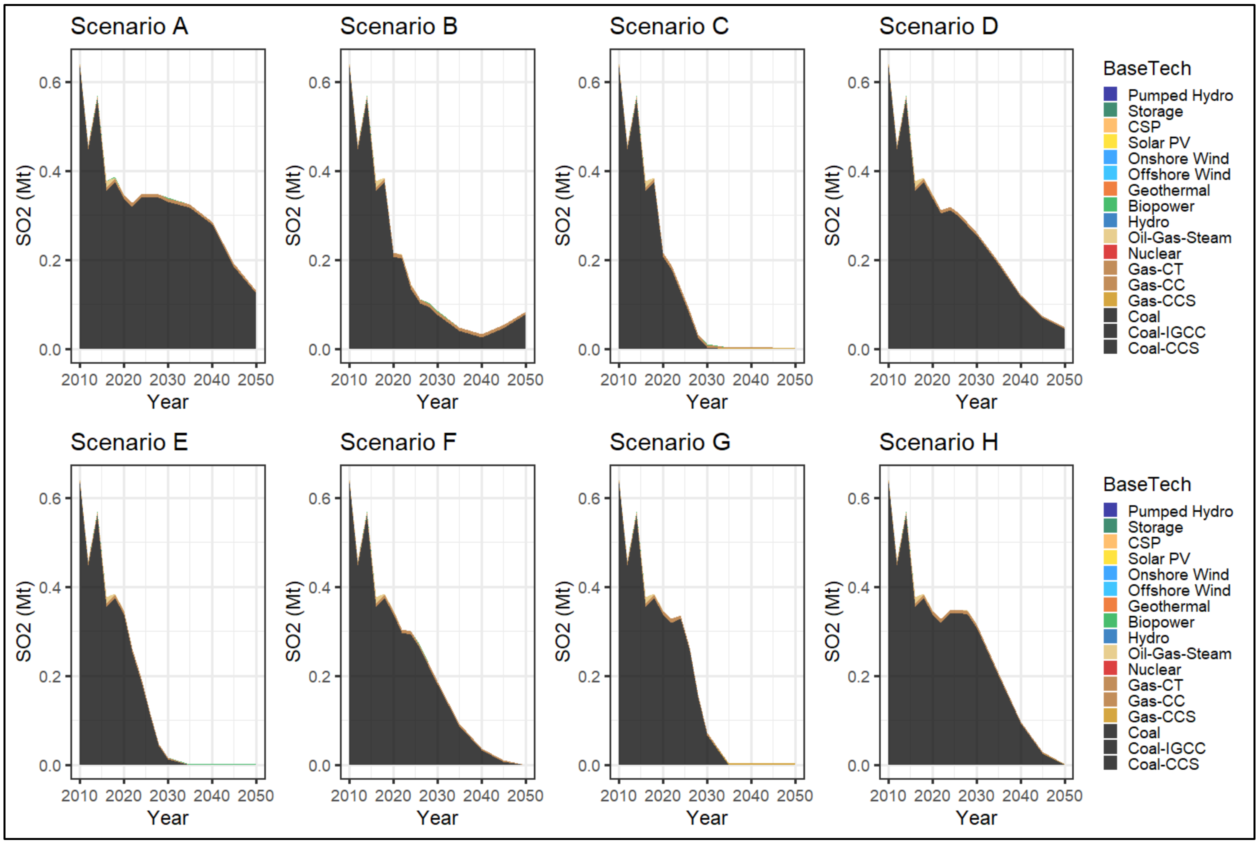

Figure S-5: National operating SO_2 emissions by technology 2010 – 2050.

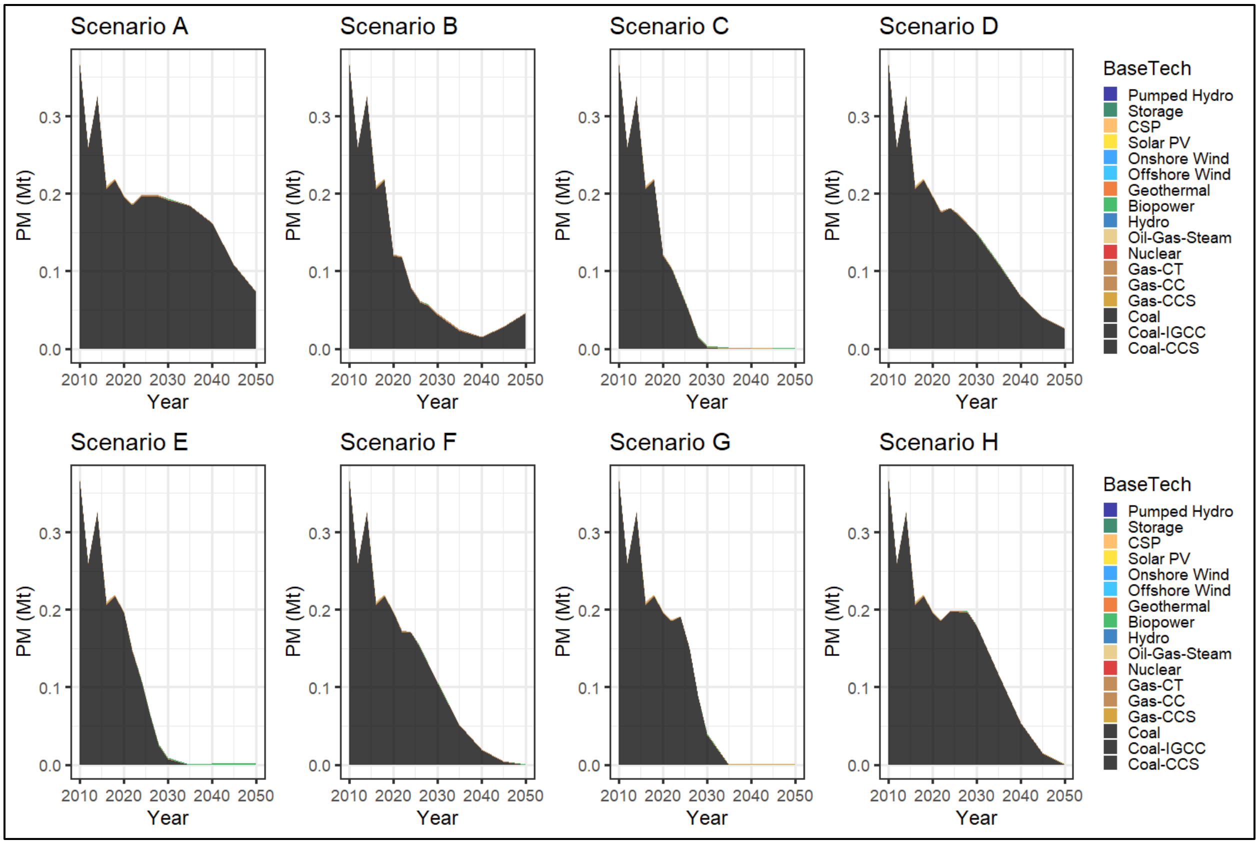

Figure S-6: National operating $PM_{2.5}$ emissions by technology 2010 – 2050.

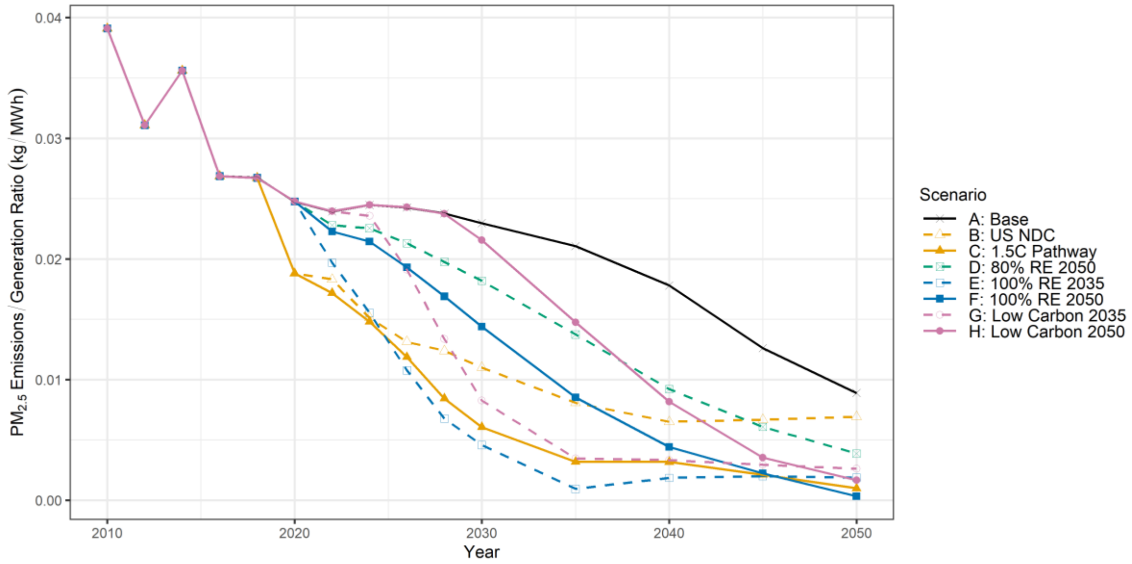

Figure S-7: Ratio between $PM_{2.5}$ emissions and generation (in kg/MWh). This shows the national emissions ratio of $PM_{2.5}$ emissions per MWh generation. We see this is a similar trend across scenarios as to the national $PM_{2.5}$ emissions (see Figure 2 of the main text).

F. Regional Air Pollution Analysis

Figures S-8 and S-9 show the NO_x and SO_2 emissions distribution in 2020, 2035, and 2050.

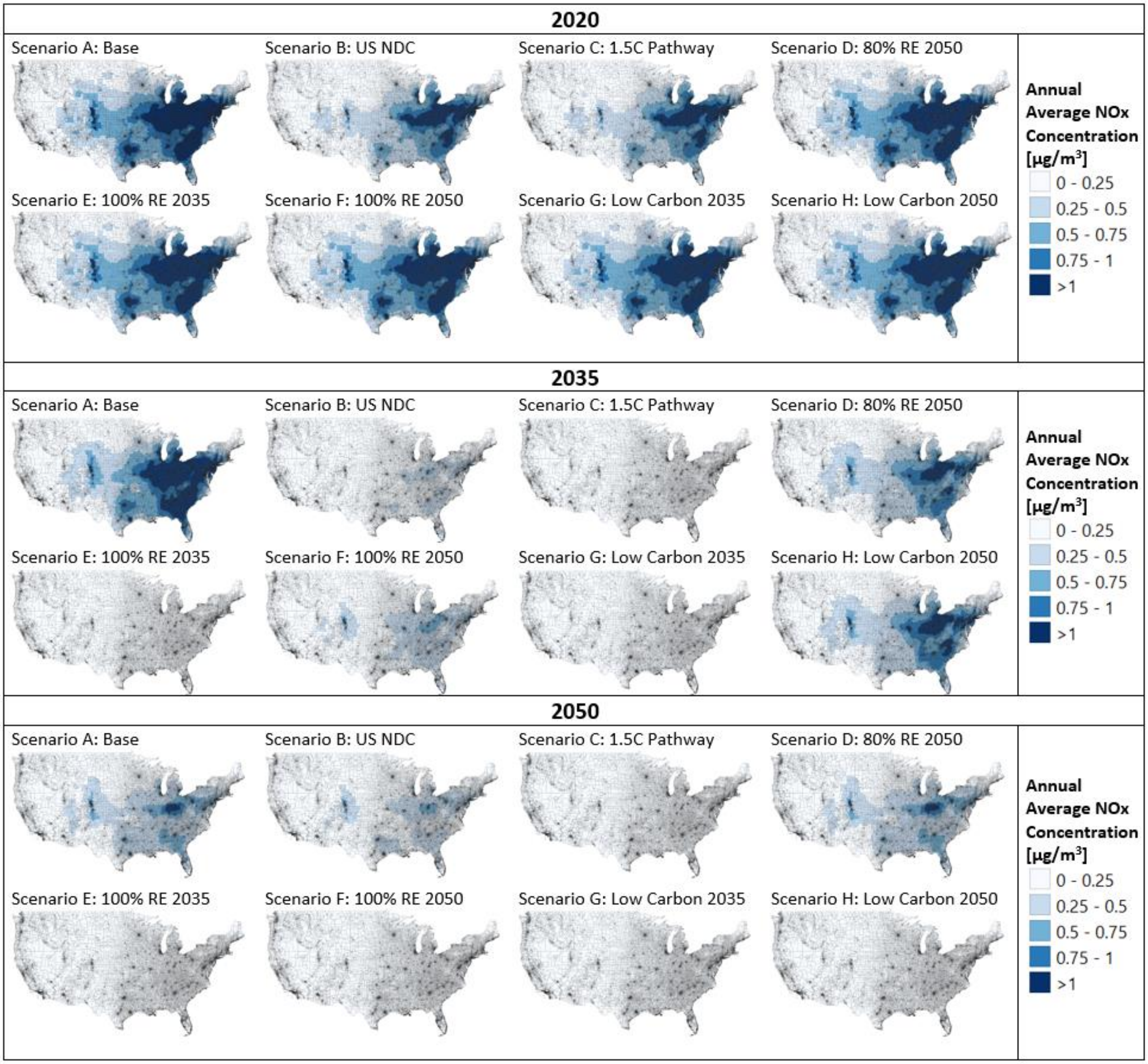

Figure S-8: Distribution of NO_x emissions 2020, 2035, and 2050.

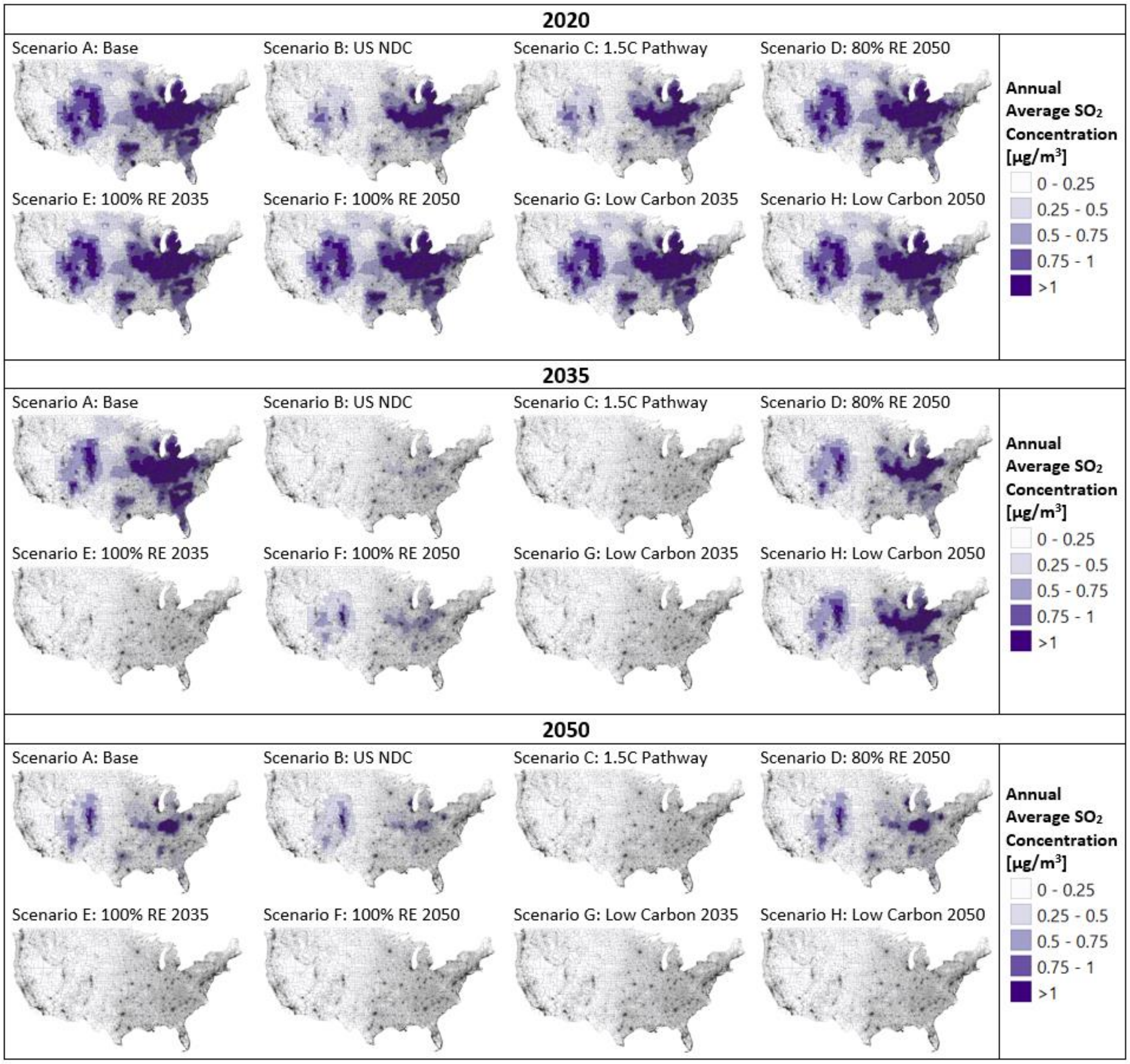

Figure S-9: Distribution of SO_2 emissions 2020, 2035, and 2050.

G. Air Pollution Equality Assessments

Here we present the air pollution equality assessments in our analysis. Table S-5 shows the groups and respective sample sizes for the equality analysis. Table S-6 shows the reductions in $PM_{2.5}$ concentrations per billion metric tons of CO_2 reduced from 2020 to 2050. A value of one indicates that the rate of change of $PM_{2.5}$ (in µg per m^3) is the same as the rate of change of CO_2 (in billion metric tons). A value of less than one indicates that the rate of reductions in CO_2 are greater than the rate of reductions in local pollutants. This ratio is useful for understanding the impact of decarbonization policies on the rate of reduction of $PM_{2.5}$ in relation to CO_2. From Table S-6 we see that Scenario D (80% renewable energy by 2050) has the highest reduction of PM2.5 compared to billion metric tons of CO_2 reduced. This means that we see the most benefit in health impacts (represented as reductions in $PM_{2.5}$ concentrations) from reaching 80% renewable energy by 2050 per billion metric tons of CO_2 reduced. Scenario A has the third highest ratio, but overall has the worst health impacts due to this scenario having the highest level of total emissions.

Figures S-10 and S-11 show the population weighted annual average NO_x and SO2 concentration across racial or ethnic groups in all scenarios 2020 – 2050. Interestingly we find that Black communities are always worse off than their racial/ethnic counterparts. Figure S-12, S-13, and S-14 show the population weighted $PM_{2.5}$, NO_x, and SO_2 concentration across all income groups and scenarios 2020 to 2050 respectively. Figure S-15 shows the population weighted $PM_{2.5}$ concentration across race and ethnicity groups for the two highest and two lowest income groups. We see that even within income groups, Black communities are burdened with the most air pollution. Figure 16 shows the absolute change of $PM_{2.5}$ concentration over race and ethnicity groups, showing that Black communities see the largest reductions over the energy transition. Figure 17 shows the percent change of $PM_{2.5}$ with respect to the starting point in 2020 across race and ethnicity groups and scenarios. We see that the percent change across race and ethnicity groups within scenarios is the same over each modeling period, indicating that starting points in 2020 impacts air pollution exposure over the energy transition. Figures S-18 and S-19 show the population weighted NO_x and SO_2 concentration respectively across scenarios and poverty groups. Figure S-20 shows the distribution of percent Black and non-Latinx white census tracts and $PM_{2.5}$ regional distribution in 2020 and 2050.

Table S-5: Equality groups and their respective sample sizes. Emission concentrations were averaged over these groups to get the population weighted average annual concentration in each group.

	Groups	Sample Size (number of census tracts)
Population in census tract below the Poverty Line (%)	[0, 10%]<br>(10%, 20%]<br>(20%, 30%]<br>(30%, 50%]<br>(50%, 70%]<br>(70%, 100%]	26,850<br>22,897<br>11,611<br>8,621<br>1,389<br>189
Median Income	[0, $25k]<br>($25k, $50k]<br>($50k, $75k]<br>($75k, $100k]<br>($100k, $125k]<br>($125k, $150k]<br>($150k,]	4,458<br>29,389<br>22,710<br>8,942<br>3,843<br>1,294<br>890

Table S-6: Ratio of reductions in $PM_{2.5}$ concentration per billion metric tons of CO_2 from 2020 to 2050 (in µg-m^{-3} per billion metric tons). The PM reductions are calculated as a population weighted annual average.

Decarbonization Scenario	Black	Non-Latinx White	Asian	Latinx/ Hispanic	Indigenous
Scenario D – 80% RE 2050	0.568	0.474	0.32	0.291	0.311
Scenario F – 100% RE 2050	0.527	0.45	0.302	0.28	0.305
Scenario A – Base	0.526	0.442	0.308	0.269	0.29
Scenario H – Low Carbon 2050	0.526	0.45	0.301	0.278	0.305
Scenario G – Low Carbon 2035	0.525	0.449	0.299	0.276	0.304
Scenario E – 100% RE 2035	0.523	0.448	0.301	0.279	0.303
Scenario C – 1.5℃	0.347	0.295	0.195	0.169	0.183
Scenario B – US NDC	0.327	0.271	0.18	0.144	0.154

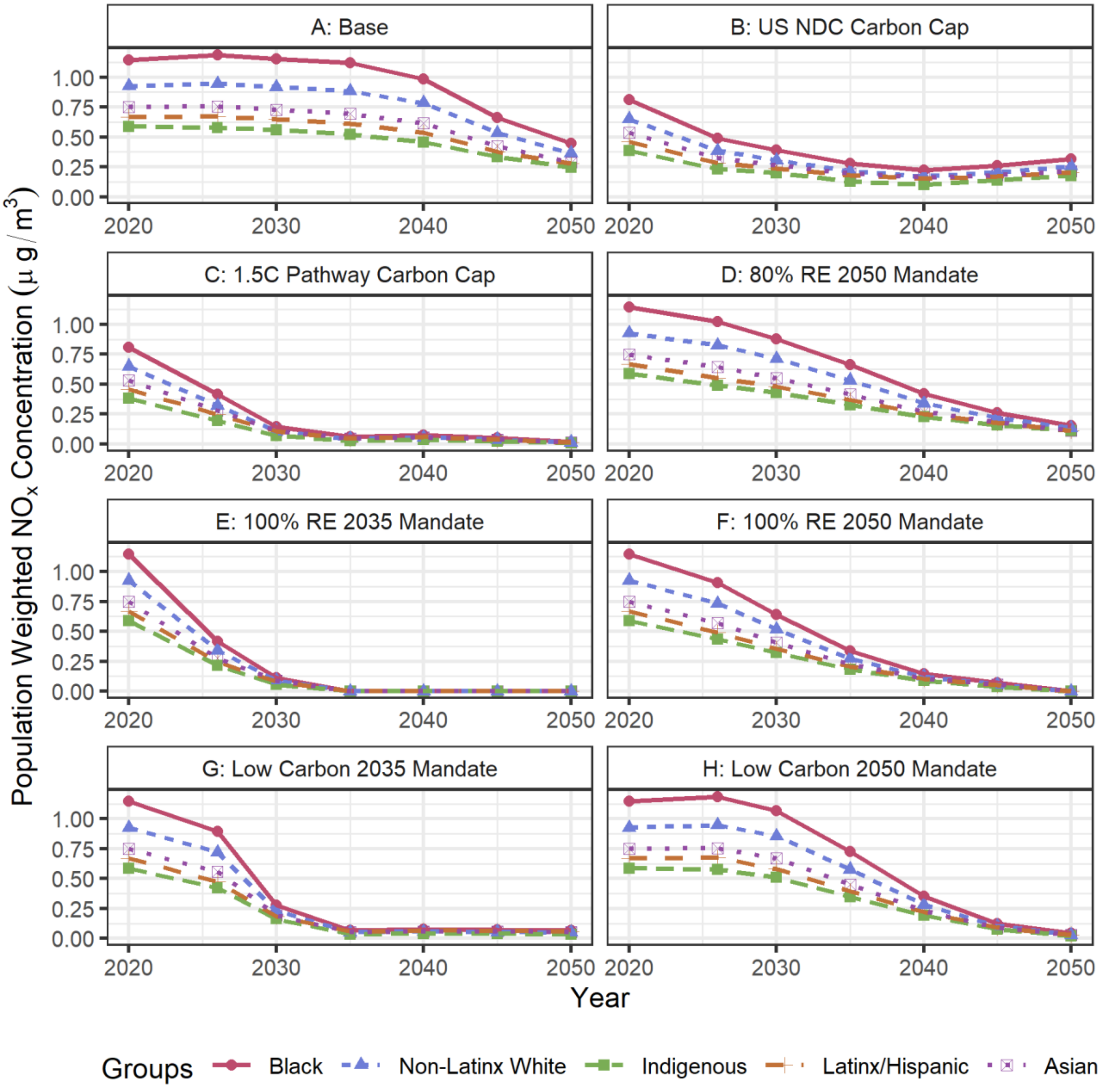

Figure S-10: Population weighted annual average NO_x concentration across race and ethnicity groups. We see black populations reside in areas with high NO_x concentrations across all decarbonization scenarios and time periods.

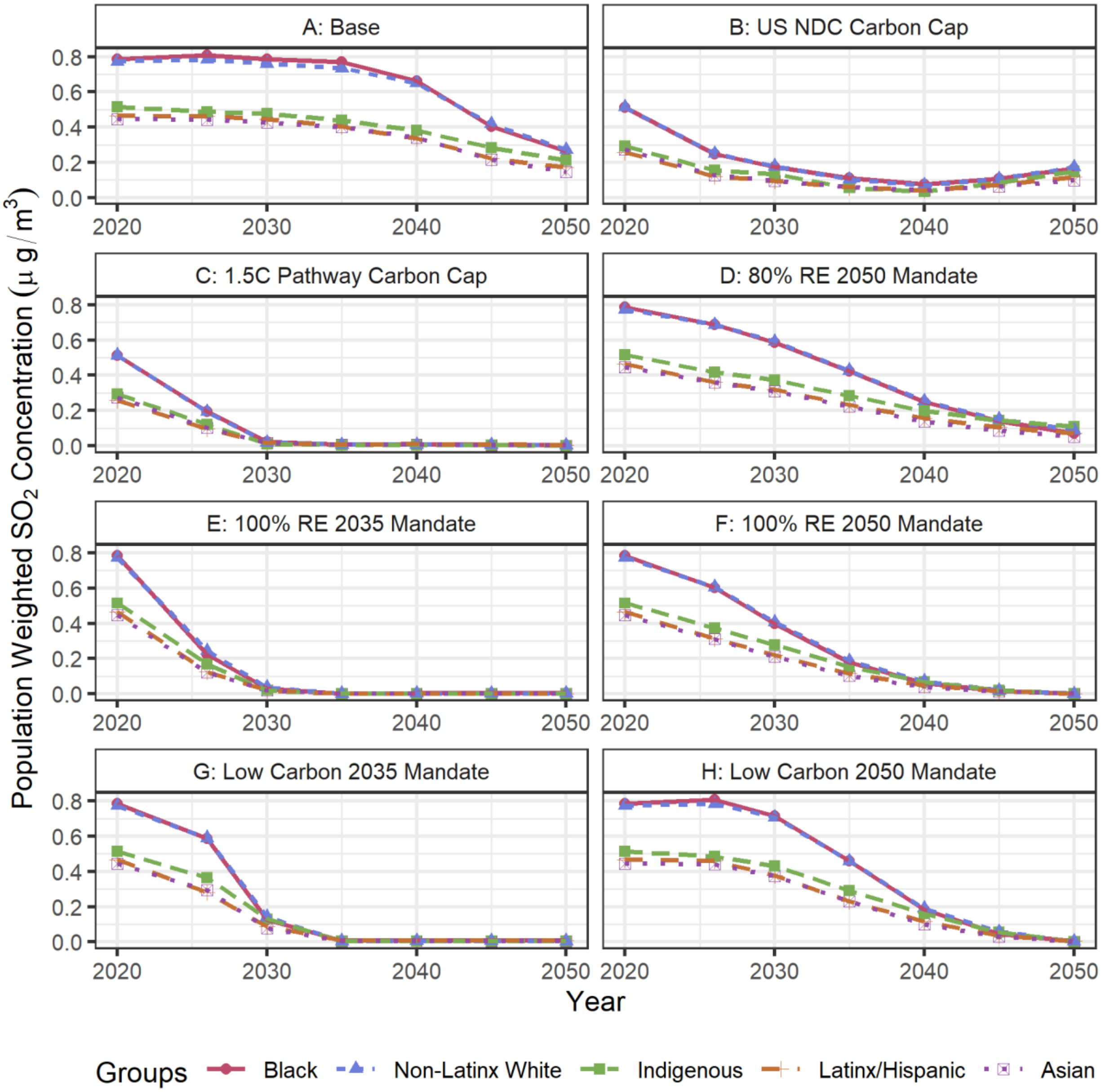

Figure S-11: Population weighted annual average SO_2 concentration across race and ethnicity groups.

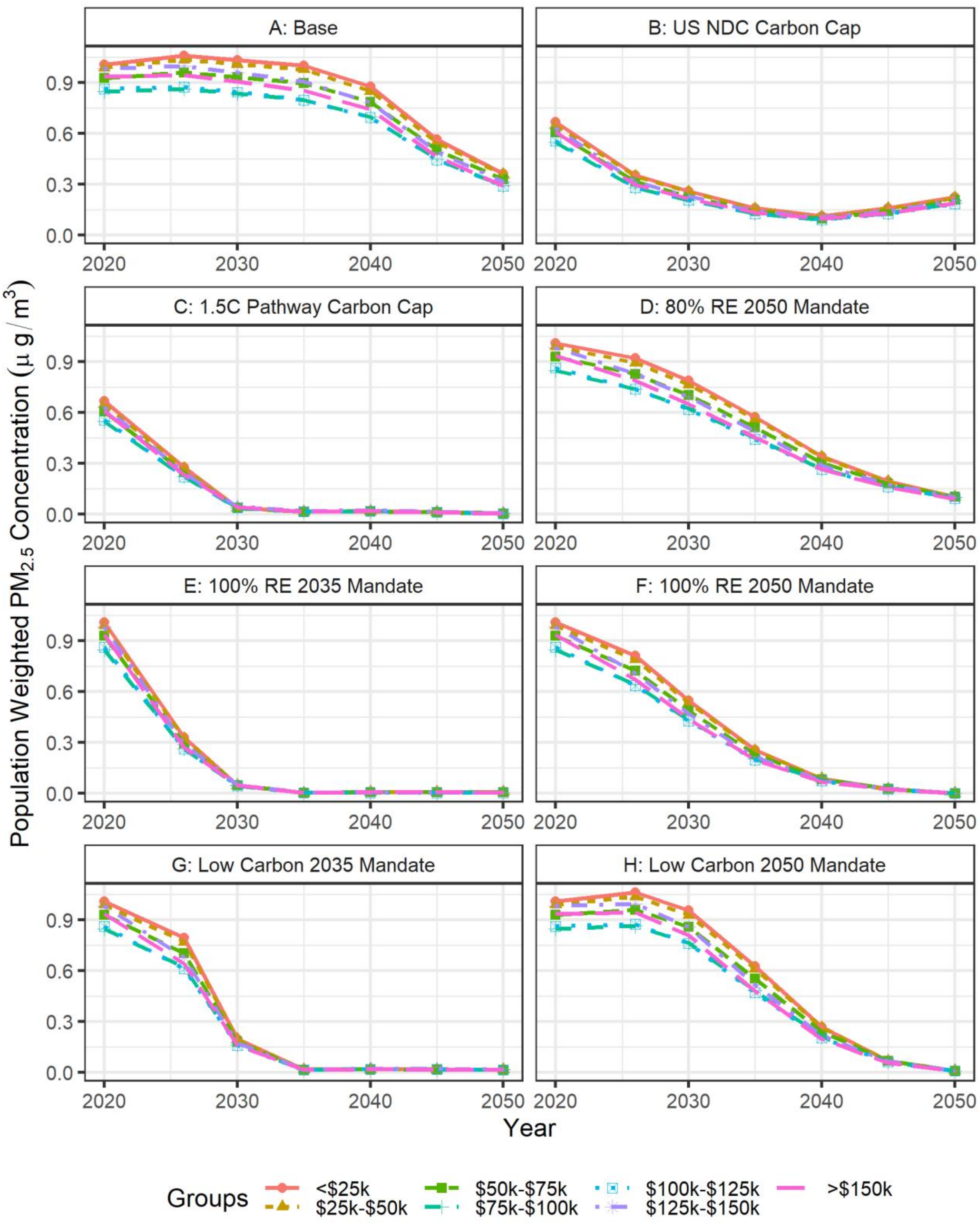

Figure S-12: Population weighted $PM_{2.5}$ concentration across all income groups and scenarios from 2020 – 2050.

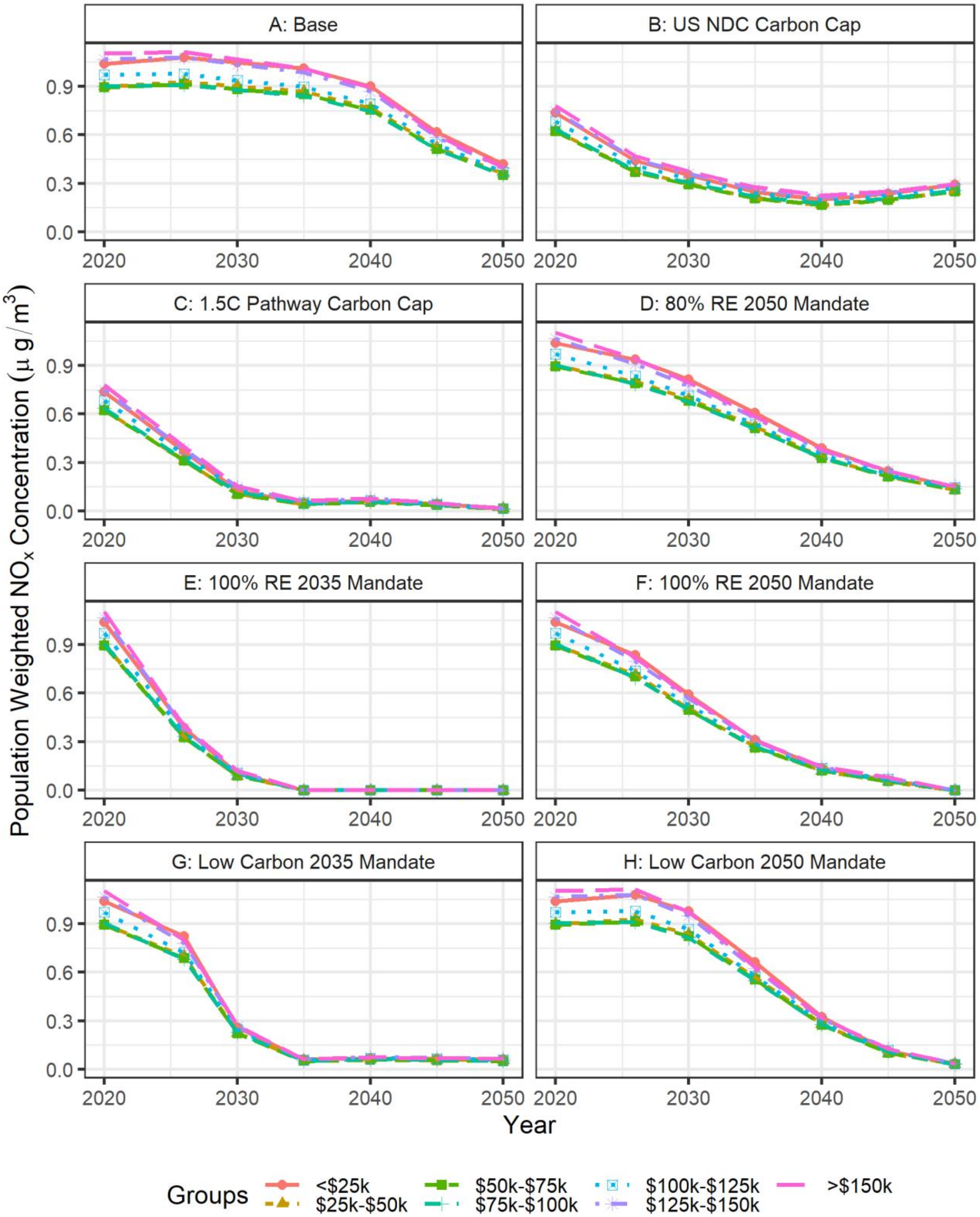

Figure S-13: Population weighted NO_x concentration across all income groups and scenarios 2020 – 2050.

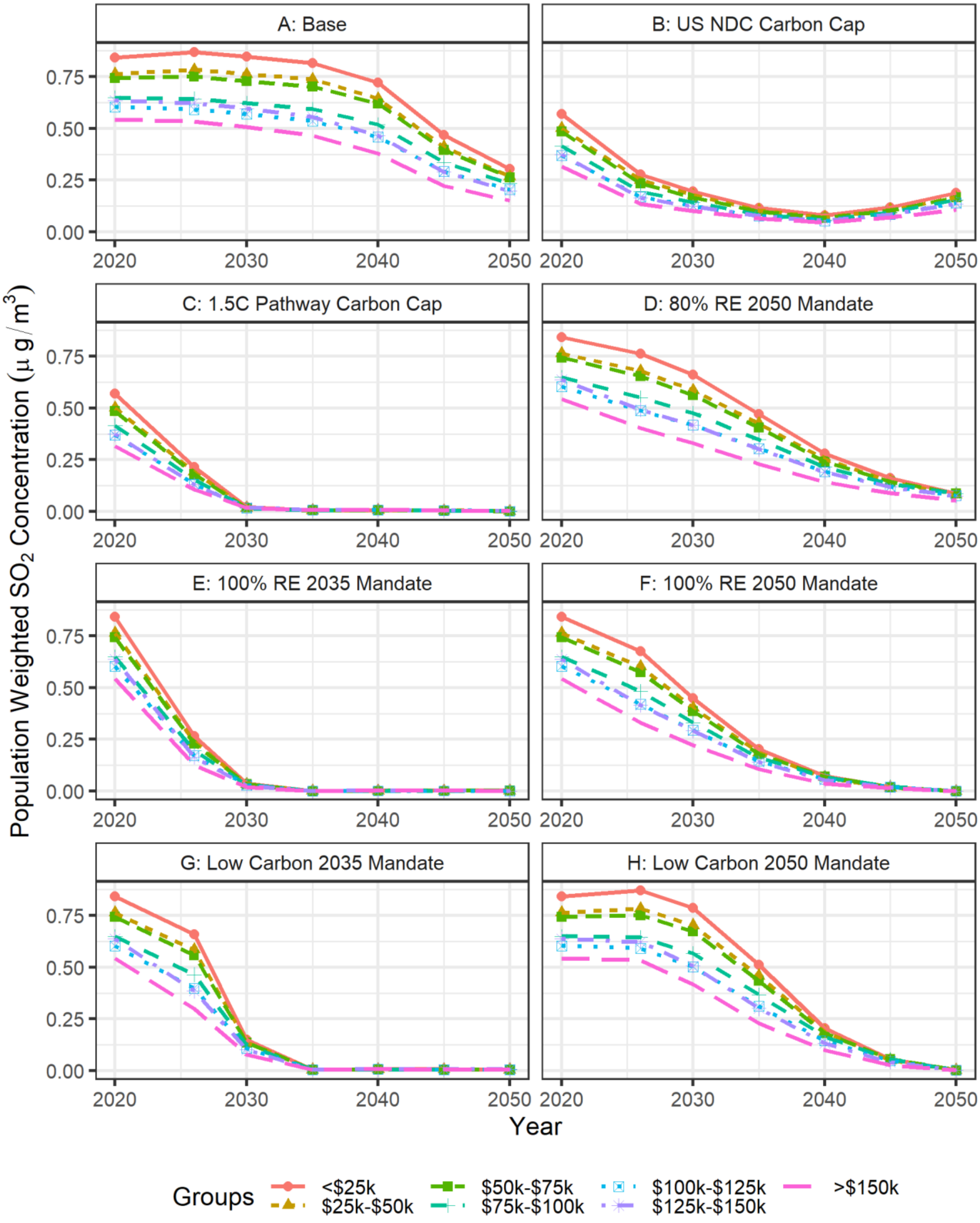

Figure S-14: Population weighted SO_2 concentration across all income groups and scenarios 2020 – 2050.

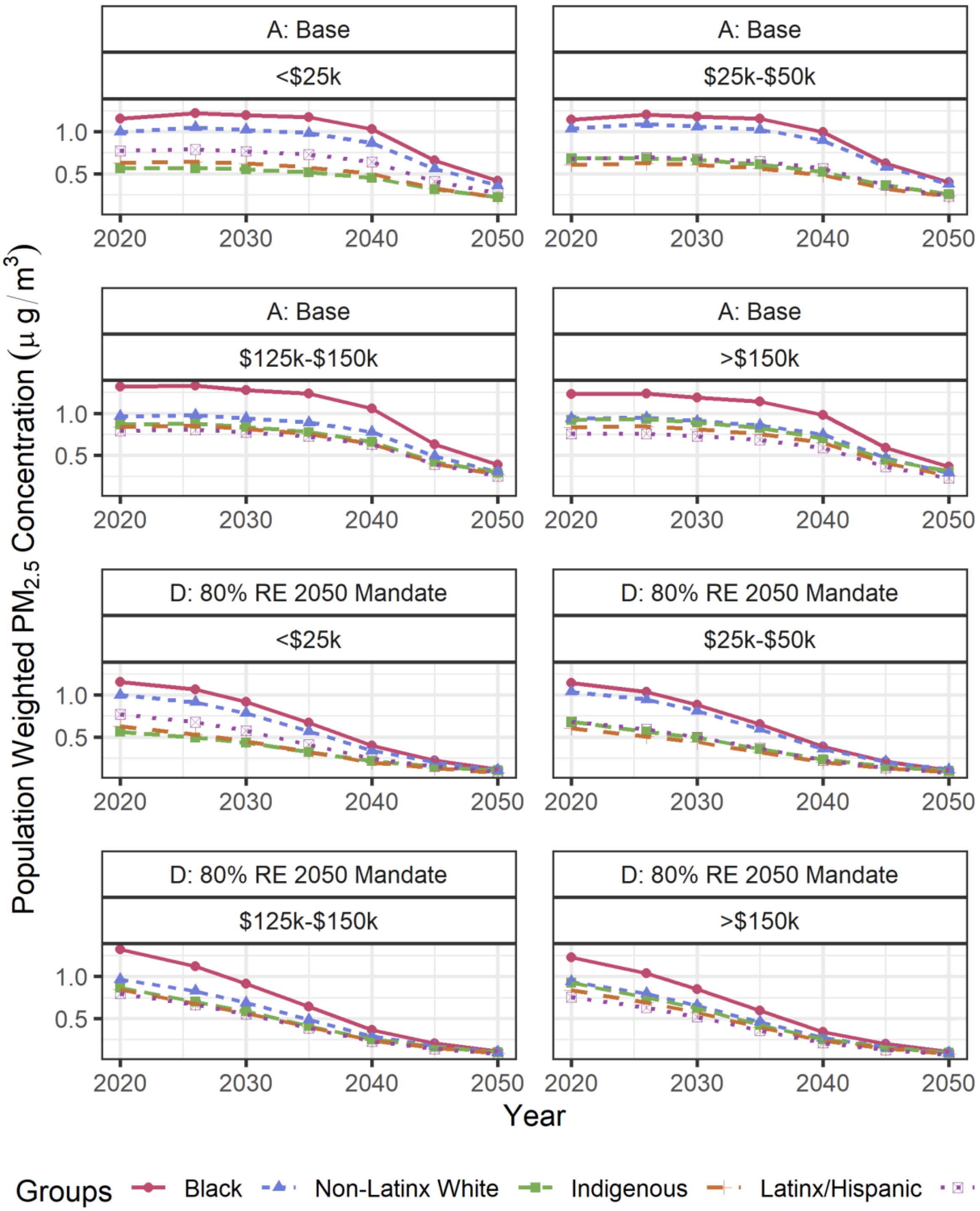

Figure S-15: Distribution of population weighted $PM_{2.5}$ concentration across race and ethnicity in the two highest ($125k-$150k and >$150k) and two lowest (<$25k and $25k-$50k) median income brackets for Scenario A (Base Case) and Scenario D (80% RE by 2050 Mandate). This shows that across income groups, Black people are exposed to the highest concentrations of $PM_{2.5}$.

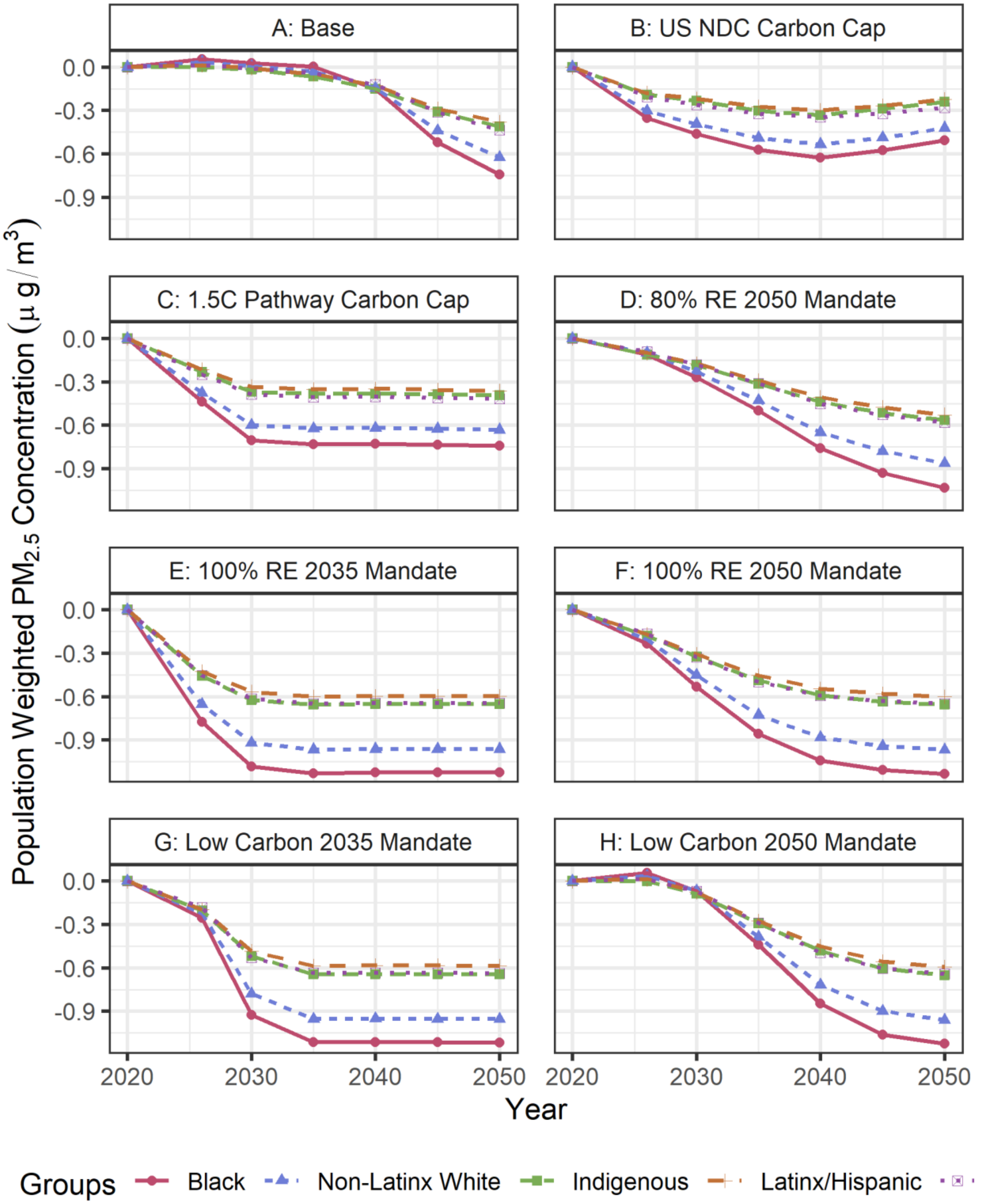

Figure S-16: Absolute changes in population weighted $PM_{2.5}$ concentrations (in μg/m^3) across race and ethnicity groups for each scenario 2020 – 2050.

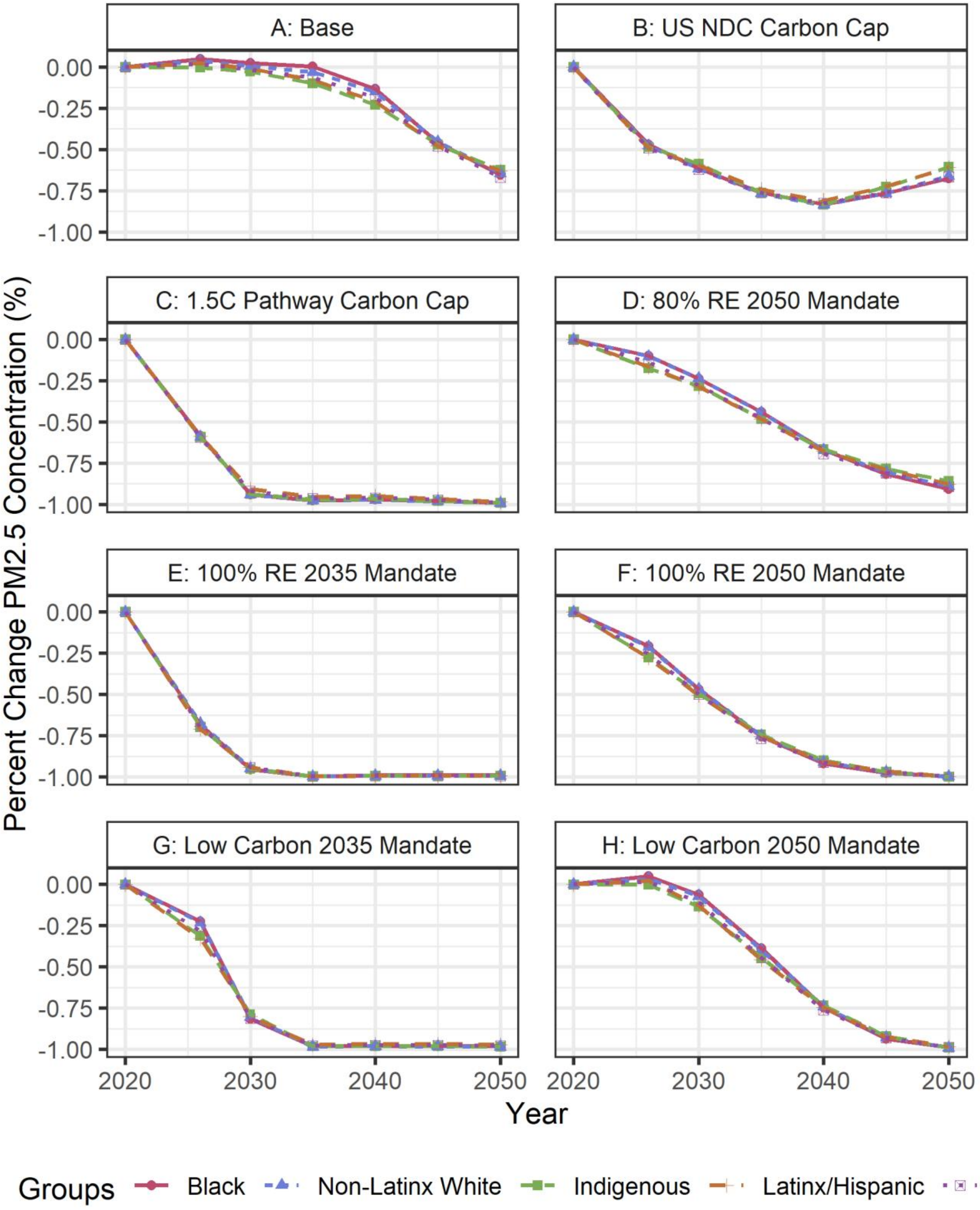

Figure S-17: Percent change of population weighted $PM_{2.5}$ concentration across race and ethnicity groups for each scenario over the energy transition 2020 – 2050.

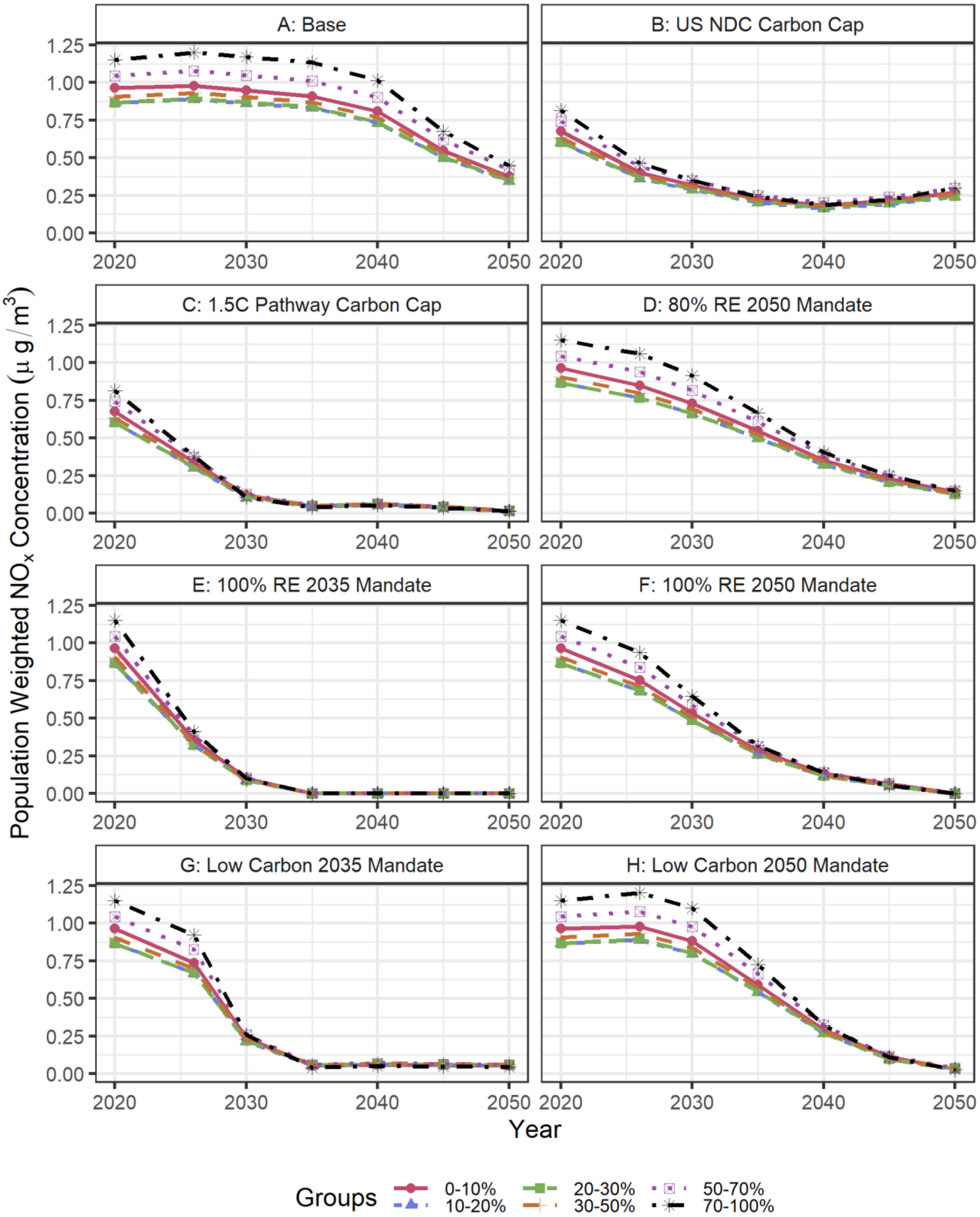

A: Base
B: US NDC Carbon Cap
C: 1.5C Pathway Carbon Cap
D: 80% RE 2050 Mandate
E: 100% RE 2035 Mandate
F: 100% RE 2050 Mandate
G: Low Carbon 2035 Mandate
H: Low Carbon 2050 Mandate
Population Weighted NOx Concentration (μ g / m3)
Year
Groups
0-10%
10-20%
20-30%
30-50%
50-70%
70-100%

Figure S-18: Population weighted NO_x concentration across poverty rate groups. We see that areas with high poverty rates reside in areas with high NO_x concentrations across all decarbonization scenarios and time periods.

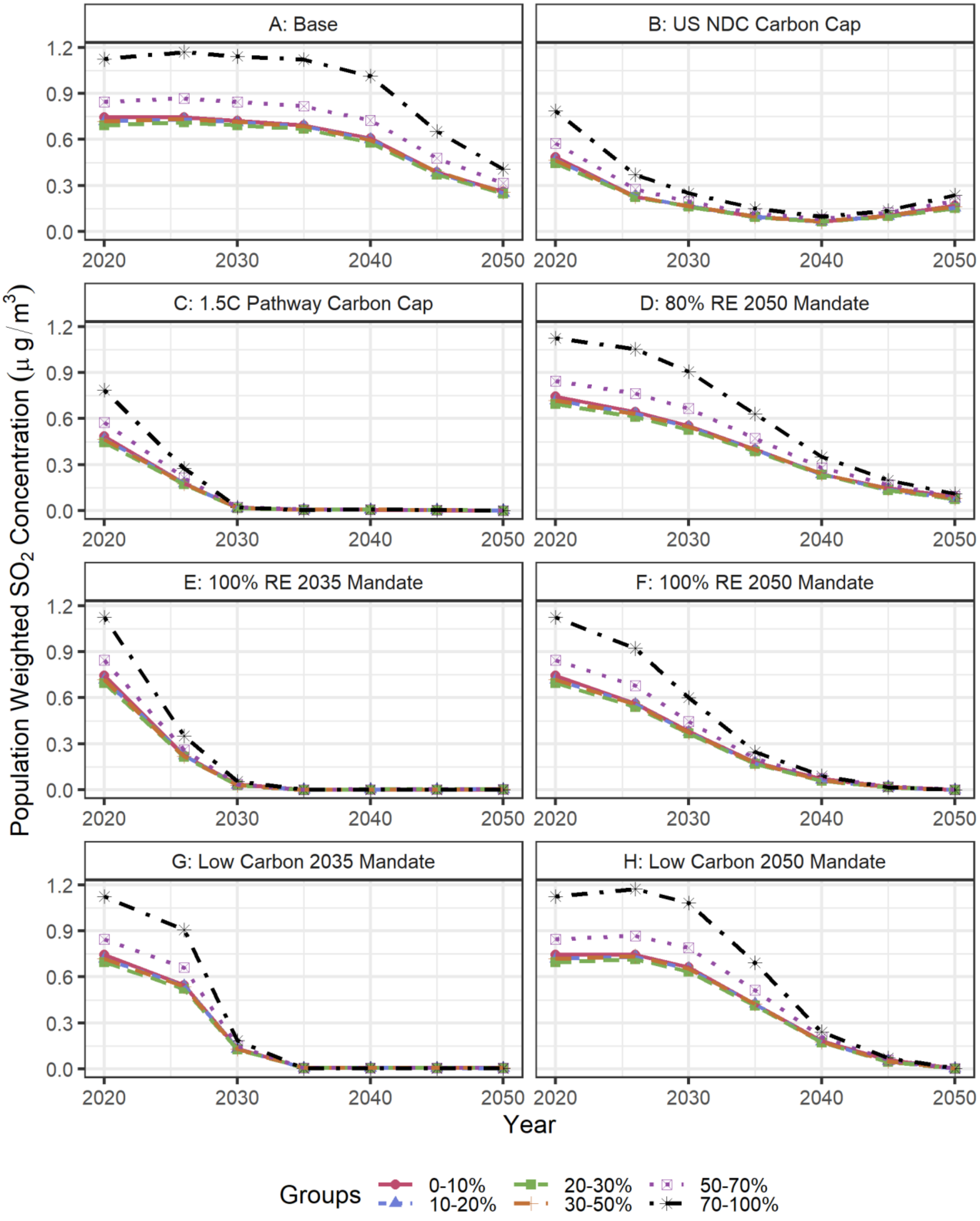

Figure S-19: Population weighted SO_2 concentration across poverty rate groups. We see that areas with high poverty rates reside in areas with high SO_2 concentrations across all decarbonization scenarios and time periods.

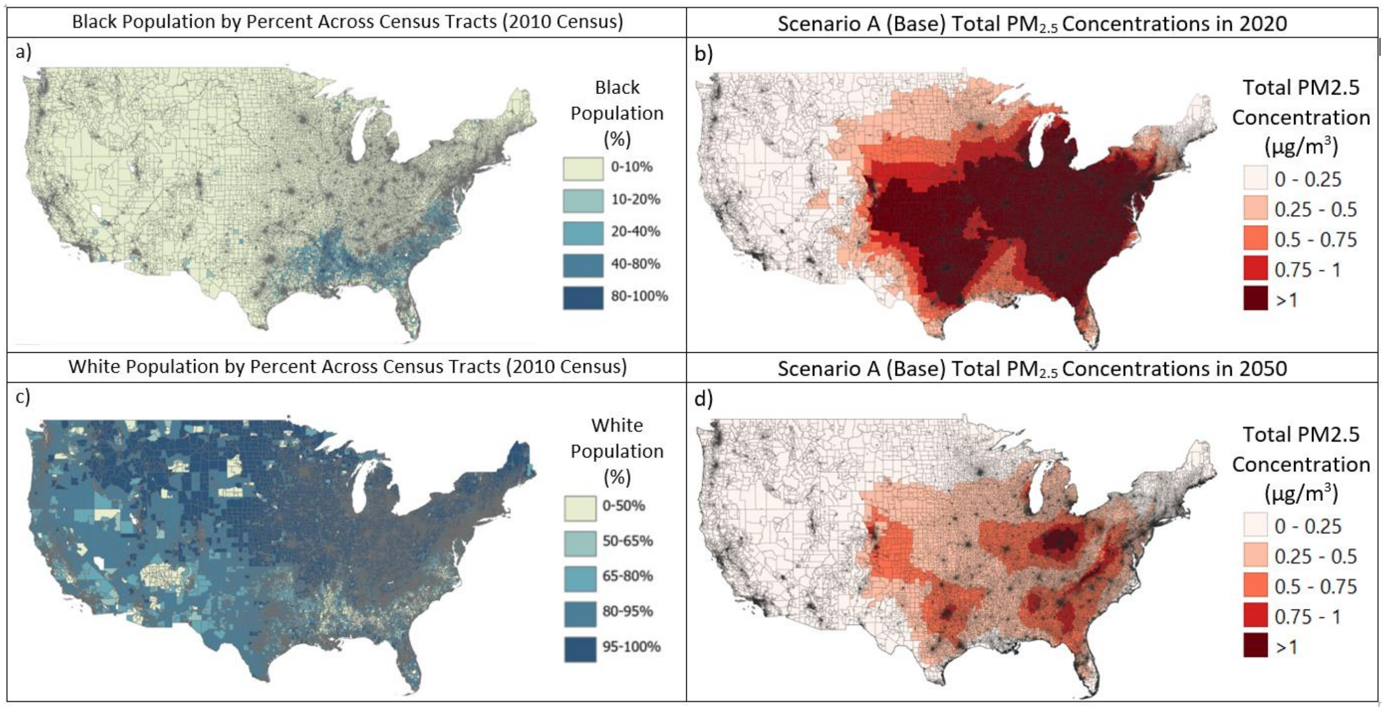

Figure S-20: Regional distribution of air pollution and racial/ethnic groups. The distribution of racial groups includes the (a) Black and (c) non-Latinx white communities by census tract. The distribution of air pollution in the Base Case (Scenario A) are shown for the years (b) 2020 and (d) 2050. We show the Black and white racial groups because these communities are exposed to the highest concentrations of $PM_{2.5}$ from the electricity sector across the entire modeling horizon in our analysis.

References

1. Cohen, S. *et al. Regional Energy Deployment System (ReEDS) Model Documentation: Version 2018*. https://www.nrel.gov/docs/fy19osti/72023.pdf. (2019).

2. US EPA. *Estimating Particulate Matter Emissions for eGRID*. (2020).

3. Tessum, C. W., Hill, J. D. & Marshall, J. D. InMAP: A model for air pollution interventions. *PLoS One* **12**, e0176131 (2017).

4. Prener, C. Areal Weighted Interpolation. https://cran.r-project.org/web/packages/areal/vignettes/areal-weighted-interpolation.html.
