# Supplementary material for: Air pollution disparities and equality assessments of US national decarbonization strategies
Source: Nat Commun. 2022 Dec 5;13:7488. doi: 10.1038/s41467-022-35098-4 (PMC9722701; doi:10.1038/s41467-022-35098-4)
Supplement: Supplementary file 3 — Reporting Summary [file 41467_2022_35098_MOESM3_ESM.pdf]

nature portfolio

Corresponding author(s): Teagan Goforth
Destenie Nock

Last updated by author(s): 11/9/2022

Reporting Summary

Nature Portfolio wishes to improve the reproducibility of the work that we publish. This form provides structure for consistency and transparency in reporting. For further information on Nature Portfolio policies, see our Editorial Policies and the Editorial Policy Checklist.

Statistics

For all statistical analyses, confirm that the following items are present in the figure legend, table legend, main text, or Methods section.

n/a	Confirmed	
☒	☐	The exact sample size (n) for each experimental group/condition, given as a discrete number and unit of measurement
☒	☐	A statement on whether measurements were taken from distinct samples or whether the same sample was measured repeatedly
☒	☐	The statistical test(s) used AND whether they are one- or two-sided<br>*Only common tests should be described solely by name; describe more complex techniques in the Methods section.*
☒	☐	A description of all covariates tested
☒	☐	A description of any assumptions or corrections, such as tests of normality and adjustment for multiple comparisons
☒	☐	A full description of the statistical parameters including central tendency (e.g. means) or other basic estimates (e.g. regression coefficient) AND variation (e.g. standard deviation) or associated estimates of uncertainty (e.g. confidence intervals)
☒	☐	For null hypothesis testing, the test statistic (e.g. F, t, r) with confidence intervals, effect sizes, degrees of freedom and P value noted<br>*Give P values as exact values whenever suitable.*
☒	☐	For Bayesian analysis, information on the choice of priors and Markov chain Monte Carlo settings
☒	☐	For hierarchical and complex designs, identification of the appropriate level for tests and full reporting of outcomes
☒	☐	Estimates of effect sizes (e.g. Cohen's d, Pearson's r), indicating how they were calculated

Our web collection on statistics for biologists contains articles on many of the points above.

Software and code

Policy information about availability of computer code

Data collection	For data collection, we used the Regional Energy Deployment System from the National Renewable Energy Lab, which is an open source capacity expansion model. We also use the Intervention Model for Air Pollution to estimate air pollution, which is also an open source model. ReEDS uses R 3.4.4, Python 3.6.5, and GAMS 30.3. ReEDS also uses the GAMS/CPLEX license for the LP solver.
Data analysis	Python 3.8.3 and pandas 1.3.4 (a Python data analysis package) were used for data analysis in this paper. ArcGIS Pro 3.0.2, QGIS 3.16.11, R 3.6.2 and ggplot2 R package were used to create figures in this paper.

For manuscripts utilizing custom algorithms or software that are central to the research but not yet described in published literature, software must be made available to editors and reviewers. We strongly encourage code deposition in a community repository (e.g. GitHub). See the Nature Portfolio guidelines for submitting code & software for further information.

Data

Policy information about availability of data

All manuscripts must include a data availability statement. This statement should provide the following information, where applicable:

- Accession codes, unique identifiers, or web links for publicly available datasets
- A description of any restrictions on data availability
- For clinical datasets or third party data, please ensure that the statement adheres to our policy

The models used in this analysis (ReEDS and InMAP) are both open-source tools. ReEDS requires R 3.4.4, Python 3.6.5, and GAMS 30.3. Python 3.8.3, ArcGIS Pro

3.0.2, QGIS 3.16.11, and R 3.6.2 were used to process data and create figures. The processed ReEDS generation outputs, regional emissions, InMAP air pollution raw outputs, InMAP air pollution processed data, and population weighted air pollution outputs data generated in this study have been deposited in GitHub database https://github.com/tgoforth27/Goforth-Nock-Air-Pollution-Inequality. The ReEDS decarbonization scenarios inputs are provided in the Supplementary Information file Section A. The census data used in this study are available at https://services.arcgis.com/P3ePLMYs2RVChkJx/arcgis/rest/services/ACS_10_14_Population_by_Race_and_Hispanic_Origin_Boundaries/FeatureServer66, and income and poverty data used in this study are available at https://services1.arcgis.com/4yjifSiIG17X0gW4/arcgis/rest/services/SES_indicators/FeatureServer67. The datasets of demographic data are shapefiles adapted from the 2010-2014 and 2011-2015 American Community Survey from the US Census Bureau respectively.

Human research participants

Policy information about studies involving human research participants and Sex and Gender in Research.

Reporting on sex and gender	n/a
Population characteristics	n/a
Recruitment	n/a
Ethics oversight	n/a

Note that full information on the approval of the study protocol must also be provided in the manuscript.

Field-specific reporting

Please select the one below that is the best fit for your research. If you are not sure, read the appropriate sections before making your selection.

☒ Life sciences ☐ Behavioural & social sciences ☐ Ecological, evolutionary & environmental sciences

For a reference copy of the document with all sections, see nature.com/documents/nr-reporting-summary-flat.pdf

Life sciences study design

All studies must disclose on these points even when the disclosure is negative.

Sample size	In our simulation, sample sizes for census tract grouping were dictated by on percentage of a given race or ethnicity lived in a census tract, median income, or percent poverty. Sample sizes of median income and poverty rates were determined by grouping these data by percentages or income that would capture heterogeneity of the demographics across census tracts and the US, as well as the large ranges of poverty and income across the US. Race/ethnicity data was grouped by reported race/ethnicity in the US census. In our study, we use the entire US population as reported in the US Census American Community Surveys.
Data exclusions	No data were excluded from the analysis.
Replication	Replicated the capacity expansion model results using the same decarbonization scenarios. The analysis on emissions, air pollution distribution, and vulnerable groups were replicated twice.
Randomization	Randomization is not relevant to our study because we are investigating the impact of energy transitions and air pollution on specific demographic groups and regions. We are looking at the entire population of the US, so randomization is irrelevant since we do not have sample groups.
Blinding	Blinding is not relevant to our study because we are investigating regional and impacts of air pollution. We do not use a statistical model, so blinding is not necessary in our analysis.

Reporting for specific materials, systems and methods

We require information from authors about some types of materials, experimental systems and methods used in many studies. Here, indicate whether each material, system or method listed is relevant to your study. If you are not sure if a list item applies to your research, read the appropriate section before selecting a response.

Materials & experimental systems

n/a	Involved in the study
☒	☐ Antibodies
☒	☐ Eukaryotic cell lines
☒	☐ Palaeontology and archaeology
☒	☐ Animals and other organisms
☒	☐ Clinical data
☒	☐ Dual use research of concern

Methods

n/a	Involved in the study
☒	☐ ChIP-seq
☒	☐ Flow cytometry
☒	☐ MRI-based neuroimaging
